# Working memory representations in visual cortex mediate distraction effects

**DOI:** 10.1038/s41467-021-24973-1

**Published:** 2021-08-05

**Authors:** Grace E. Hallenbeck, Thomas C. Sprague, Masih Rahmati, Kartik K. Sreenivasan, Clayton E. Curtis

**Affiliations:** 1 Department of Psychology, New York University, New York, NY USA; 2 Department of Psychological and Brain Sciences, University of California, Santa Barbara, CA USA; 3 Center for Neural Science, New York University, New York, NY USA; 4 Division of Science and Mathematics, New York University Abu Dhabi, Abu Dhabi, UAE

**Keywords:** Attention, Working memory, Striate cortex

## Abstract

Although the contents of working memory can be decoded from visual cortex activity, these representations may play a limited role if they are not robust to distraction. We used model-based fMRI to estimate the impact of distracting visual tasks on working memory representations in several visual field maps in visual and frontoparietal association cortex. Here, we show distraction causes the fidelity of working memory representations to briefly dip when both the memorandum and distractor are jointly encoded by the population activities. Distraction induces small biases in memory errors which can be predicted by biases in neural decoding in early visual cortex, but not other regions. Although distraction briefly disrupts working memory representations, the widespread redundancy with which working memory information is encoded may protect against catastrophic loss. In early visual cortex, the neural representation of information in working memory and behavioral performance are intertwined, solidifying its importance in visual memory.

## Abstract (web summary)

The relative roles of visual, parietal, and frontal cortex in working memory have been actively debated. Here, the authors show that distraction impacts visual working memory representations in primary visual areas, indicating that these regions play a key role in the maintenance of working memory.

## Introduction

Higher cognition depends on working memory (WM), the ability to maintain and perform operations on internal representations of information no longer available in the environment. Early studies focused on a primary role of prefrontal cortex in supporting WM representations based on sustained activation over delay periods^1–4^. However, recent studies have repeatedly demonstrated that the contents of WM can be decoded from the multivariate patterns of activity in early visual cortex^5–11^. This putative dichotomy between regions coordinating WM processes, located in a frontoparietal network, and regions storing remembered information, located within the sensory cortex, has led to the development of the sensory recruitment model of WM^1,5,6,9,11–17^. This model hypothesizes that feedback signals from frontoparietal cortex recruit the mechanisms used for perceptual encoding in sensory cortex for precise memory storage.

Yet, the idea that visual cortex plays a critical role in working memory continues to be met with skepticism^18–20^, and major aspects of the sensory recruitment model remain underspecified. For instance, it is unclear how neural circuitry in early visual cortex can simultaneously maintain WM representations while encoding incoming percepts^18^. To address this, several recent studies have evaluated how WM representations might be maintained in the presence of behaviorally irrelevant sensory distraction. In each of these studies, participants remembered a visual stimulus over a delay while simultaneously viewing an irrelevant visual stimulus. The results from these studies are highly inconsistent; WM representations in visual cortex were eliminated^21^, spared (Rademaker et al.^22^; exp. 1), partially disrupted (Rademaker et al.^22^; exp. 2), or biased^23^ as a result of distraction. Even within studies, alterations to distractor properties result in changes in the information contained in visual cortical representations (compare exps. 1 and 3 in refs. ^21^; exps. 1 and 3 in Rademaker et al.^22^). Because visual cortex does not always contain a representation of remembered information during distraction when behavioral performance is not impacted, its role in WM representation has been considered unimportant^18,20^.

In contrast, the contents of WM remain decodable from parietal cortex activation patterns even when those in visual cortex are lost^20–23^, and in some cases the parietal WM representations appear to be stored in a different format from that used to represent sensory information (Rademaker et al.^22^, exp. 2). Based on these results, one might conclude that parietal cortex, and not visual cortex, maintains distractor-resistant memory representations. But, in addition to the inconsistency of the results in visual cortex, several outstanding issues prevent such a conclusion. For instance, behavioral performance on WM tasks can be biased by distracting stimuli, suggesting that interference between the mechanisms used for perceptual encoding and memory storage may underlie these effects^24–28^. In these behavioral studies, only distracting stimuli that are closely matched to remembered stimuli impact behavioral recall performance (e.g., viewing an irrelevant oriented grating while remembering an oriented grating). Additionally, maintaining information in visual WM impacts visual perception and visual motor selection in a manner consistent with low-level sensory interference^29,30^. However, previous studies have not been able to link behavioral evidence of distraction to distortions in encoded WM representations, as distraction either had no impact on behavior^21,22^ (exp. 1) or only a group-level worsening^22^ (exp. 2) or biasing of WM performance^23^ following distraction. If the neural representation of WM in visual cortex is disrupted by distraction while behavior is spared, then its role in WM is likely to be very limited, for instance to artificial laboratory conditions with simple blank retention intervals. On the other hand, if biases in WM performance were predicted by biases in neural WM representations in some regions, this would demonstrate a crucial role for the WM representations carried by those regions. Currently, critical evidence linking behavioral performance on individual WM trials and the neural representation of information within WM is lacking.

Here, we test the hypothesis that distractor-induced distortions in WM may stem from corresponding distortions in the most critical population-encoded WM representations in the visual and association cortices. Our study focuses on spatial WM because spatial locations held in WM can be robustly and reliably decoded from the activity of retinotopically organized visual field maps spanning visual, parietal, and frontal cortices^8,10,16,31^—even at the single-trial level^32^—maximizing our ability to detect changes in memory representations as a function of distraction. We use a memory-guided saccade task for precise quantification of WM precision, bias, and response time, while enabling contact with macaque studies using similar tasks^3,33,34^, reviewed in Curtis and Sprague^35^. Participants precisely maintain the position of a single WM target over a 12 s delay period. Distractors placed at controlled geometric distances from the WM targets provide a feedforward stimulus drive while requiring a voluntary withdrawal of attention from the WM target while participants make a difficult perceptual discrimination. We reason that such a distractor should have effects both at lower (e.g., visual cortex) and higher (e.g., association cortex) levels impacting both memory storage and control functions. These task features enable the measurement of WM representations before, during, and after distraction along with conjoint distractor representations. Here, we show that distraction induces a brief dip in the fidelity of WM representations in visual, parietal, and frontal cortex. However, the WM representations quickly recover before the end of the delay period. Critically, the spatial errors in the decoded WM representations in visual cortex, but not in parietal cortex, predict trial-by-trial biases in memory, demonstrating that WM reports may depend on the read-out of these specific WM representations. Together, these results point to a critical role for sensory regions in WM maintenance, supporting a key prediction of the sensory recruitment model.

## Results

### Attention drawn to a distractor impacts working memory performance

**Fig. 1:**
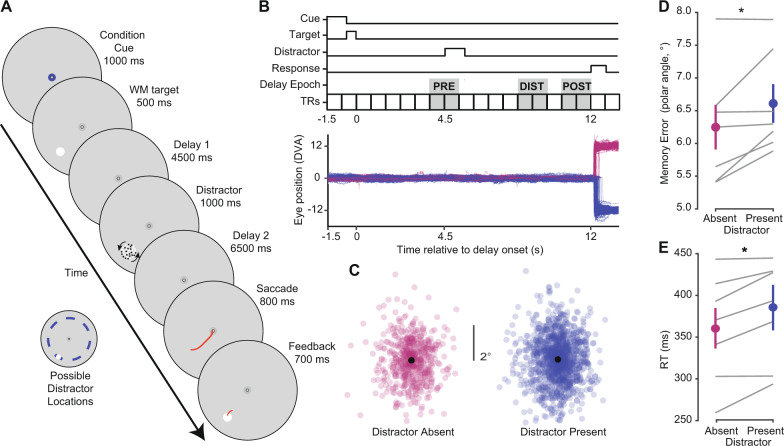
Attending a distractor stimulus impairs working memory performance. **A** Participants (*n* = 7) performed a memory-guided saccade task while brain activity and gaze were recorded inside the scanner. Each trial began with a condition cue, reliably indicating whether a distractor would appear on that trial (70% of trials) or not (30%). On each trial, participants maintained the precise spatial position of a briefly presented visual target (12° eccentricity, random polar angle) over an extended 12 s memory delay. At the end of the delay, they executed a memory-guided saccade to the remembered position. The memory target was then re-presented, and participants fixated this location before returning to central fixation. During distractor-present trials, participants discriminated whether dots presented within a 2° diameter aperture were rotating clockwise or counterclockwise with a button press. Across runs, motion coherence was varied to achieve ~75% correct performance (actual mean = 73%). The distracting stimulus could appear within one of seven position bins (24° polar angle wide) around the screen relative to the WM target, evenly presented across trials, denoted by blue intervals relative to an example WM target position (inset). **B** Timing of task events and example gaze data. Top: trial events (start of delay, distractor, and response) were synchronized to the beginning of 750 ms imaging volumes. For subsequent fMRI analyses, we defined three trial epochs for further analyses (see below, Figs. 5–7) assuming ~4 s hemodynamic delay (PRE: volumes before distractor, DIST: volumes during distractor, POST: volumes after distractor). Bottom: eye-trace of all trials of each condition for an example participant (p02). Eye position eccentricity is plotted as a function of time; distractor-absent trials are plotted with positive values, and distractor-present trials are plotted with negative values. Note that gaze remains at fixation during distraction keeping the retinal position of the memory target constant. **C** Aligned final saccadic endpoints (all participants) for trials in which distractors were absent or present. All endpoints are aligned by rotating to a common spatial position (along the horizontal meridian at 12° eccentricity). **D** Memory error (standard deviation of the polar angle of saccade endpoints) varied with distractor presence (*t*-test, two-tailed, *p* = 0.039). Gray lines show individual participants (*n* = 7); colored circles show group mean (error bars reflect ±SEM). **E** Response time also varied based on distractor presence (*t*-test, two-tailed, *p* = 0.011). Colored circles show group mean (error bars reflect ±SEM); Gray lines show individual participants (*n* = 7). Analysis of behavioral performance across individual distractor location bins shown in Supplementary Fig. 1.

Participants performed a memory-guided saccade task in which they precisely remembered the location of a target on each trial (12° eccentricity, random polar angles; Fig. 1A, B). On 70% of trials, a distractor task occured in the middle of the memory delay where participants reported the direction of rotational motion of a random dot motion stimulus. To test our main hypothesis, we performed a series of analyses designed to estimate the effect of distraction on WM. First, we asked if the distractor impacted the quality of WM (Fig. 1C, D). Memory-guided saccades were less precise on distractor-present compared to distractor-absent trials (Fig. 1D, *p* = 0.039; two-tailed *t*-test). Similarly, the initiation of memory-guided saccades was slower on distractor-present trials (Fig. 1E; *p* = 0.011; two-tailed *t*-test). The magnitude of these distractor-induced perturbations to memory-guided saccades were similar to those induced by transcranial magnetic stimulation applied to frontal and parietal cortex^36^. Moreover, as previous work indicates that distractors that are more similar to memoranda are most disruptive^24–28,37^, we tested whether the impact of distraction depended on how close the distractor was to the WM target. These follow-up analyses found no significant evidence that WM precision (*p* = 0.310; one-way repeated-measures ANOVA) or response times (*p* = 0.149; one-way repeated-measures ANOVA) varied by their locations relative to the WM target on distractor-present trials (Supplementary Fig. 1a). Overall, we observed that a brief behaviorally relevant distractor impacted WM performance. This is similar to previous studies that reported unattended distractors interfered with WM performance (refs. ^22–24^, exp. 2; but see Rademaker et al.^22^, exp. 1, and Bettencourt and Xu^21^) for reports that distraction has no effect on WM performance).

### Spatially selective BOLD activations persist during WM and respond to distraction

**Fig. 2:**
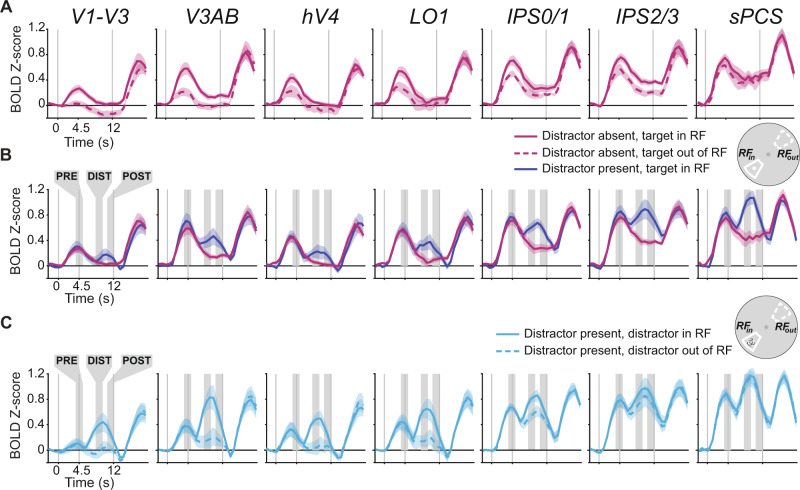
BOLD responses sorted by voxel RF position during WM delay period. **A** During distractor-absent trials, the average (±SEM) amplitude of BOLD responses was greater in voxels whose receptive fields—estimated using nonlinear population receptive field mapping^36^, see Fig. 3A for an example hemisphere)—aligned with the WM target (RF_in_) compared to when the target was 180° away from voxels’ receptive fields (RF_out_). The inset to the right depicts an example of the RF_in_ and RF_out_ regions of the visual field with respect to the WM target location (see Methods section for more details). The amplitudes of persistent activity increased moving anterior in the dorsal stream ROIs from early visual cortex (V1–V3; V3AB) to parietal cortex (IPS0/1) to frontal cortex (IPS2/3), while the spatial selectivity (difference between RF_in_ and RF_out_) decreased. Data from ventral (hV4) and lateral retinotopic regions (LO1) is also included for completeness. Time series were baseline-corrected by removing the mean activation from −2.25 to 0 s prior to delay period onset from each time series. **B** During distractor-present trials, we observed an additional phasic response time-locked to the distractor onset across all ROIs. **C** To further illustrate the distractor response, we averaged the BOLD responses in voxels whose RFs were aligned to the distractor position, regardless of the position of the WM target. The phasic responses were more robust in voxels with RFs that matched (RF_in_) compared to opposite to the distractor (RF_out_). The shaded areas denote the pre-distractor, distractor, and post-distractor epochs that are the target of later analyses. Results for individual ROIs shown in Supplementary Fig. 2, along with visualized voxel RF parameters, and all *p*-values are available in Supplementary Table 1.

All fMRI analyses focused on retinotopic regions of interest identified using maps of polar angle and eccentricity computed from independent retinotopic mapping data using a compressive spatial summation voxel receptive field model^36^; see below). We analyzed data from all retinotopic ROIs previously reported in fMRI studies of visual WM and distraction (V1, V2, V3, V3AB, IPS0, IPS1, IPS2, IPS3, sPCS, LO1, hV4;^21,22^), and combined regions which share a foveal confluence (V1, V2, and V3; IPS0 and IPS1; IPS2 and IPS3;^36,38,39^). To index WM-related sustained activation within voxels tuned to remembered locations, we took advantage of the receptive field parameters and averaged BOLD responses across voxels in each visual field map whose receptive field matched that of the WM target location (RF_in_). We chose RF_in_ voxels with eccentricities between 2° and 15° and polar angles within 15° on either side of the WM target’s polar angle. For comparison, we also averaged BOLD responses in the voxels with receptive fields 165–195° opposite the WM target (RF_out_) using the same range of eccentricity limits. Focusing on the distractor-absent trials, we saw two patterns across the visual field map ROIs that can be seen in Fig. 2A. First, the amplitude of persistent activity during the delay period increases moving anterior in the dorsal stream ROIs from early visual cortex (V1–V3; V3AB) to parietal cortex (IPS0/1, IPS2/3) to frontal cortex (sPCS), consistent with previous reports^5,6,8,10,14,31^. Second, the spatial selectivity of the persistent activity varied among the ROIs, which is apparent when comparing the RF_in_ and RF_out_ responses. Note how the amplitudes of delay period activity between the RF_in_ and RF_out_ conditions diminish from early visual cortex to parietal cortex to frontal cortex. These differences are consistent with the increasing size of receptive fields of neurons as one moves up the visual hierarchy from early visual cortex to parietal and frontal cortex^36,38,40^. We quantified these effects by testing whether delay-period activation (averaged over the period spanning 3.75 to 12 s after delay period onset) differed across position-sorted voxels and ROIs with a 2-way repeated-measures ANOVA against a shuffled null distribution with factors of ROI and RF condition (RF_in_ vs. RF_out_). The 2-way interaction was significant (*p* = 0.01), as were each of the main effects of ROI (*p* < 0.001) and RF (*p* < 0.001; individual ROI statistics in Supplementary Table 2). On distractor-present trials, the attended distractor evoked a phasic response in all ROIs, but this response was especially strong in parietal and frontal cortex (Fig. 2B). Finally, we averaged the BOLD responses in voxels whose RFs were aligned to the distractor position, regardless of the position of the WM target, to better visualize visually evoked responses associated with the distractor (Fig. 2C). The phasic distractor responses were robust in voxels with RFs that matched (RF_in_) compared to opposite to the distractor (RF_out_), especially in early visual cortex. Next, we used patterns of BOLD responses across entire ROIs to model how WM targets are encoded within the population activity of each ROI, and how distraction may disrupt such encoding across the duration of each trial.

### Reconstructed population response encodes WM targets and distractors

**Fig. 3:**
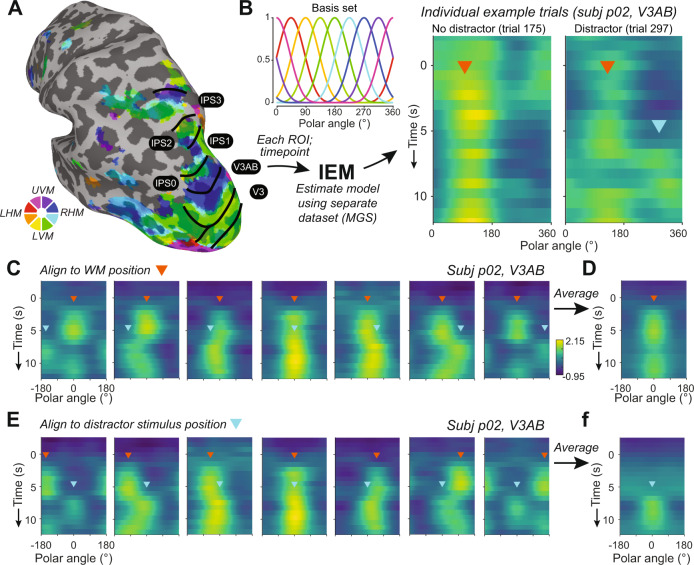
IEM-based reconstruction of WM and distractor representations. **A** Each participant underwent retinotopic mapping to define ROIs in visual, parietal, and frontal cortex (V1–V3, V3AB, hV4, LO1, IPS0-3, and sPCS). Example hemisphere and participant shown (p02, LH). Each voxel’s time course is fit with a receptive field model^36^. Color depicts preferred polar angle; thresholded at *R*^2^ ≥ 10%. **B** We estimated an inverted encoding model (IEM) for polar angle for each participant and ROI using a dataset reserved for this purpose (single-item memory-guided saccade task, 3–4.5 h/participant). Because we used a fixed encoding model^41,42^, reconstructions across different timepoints and task conditions can be directly compared to one another. Each timepoint of the spatial distractor dataset was reconstructed using this independently estimated model, and each row shows a reconstruction over polar angles computed as the sum of basis functions weighted by the corresponding reconstructed channel response (see refs. ^10,43^. Two example trials shown, and trials are not aligned to a common position. **C** In the example participant, reconstructions were aligned based on WM target positions (orange triangle), and separately averaged for each distractor bin position (cyan triangle at onset time). Note that both target and distractor representations can be seen in the reconstructions. **D** WM target reconstruction averaged over all distractor location bins. Because distractors are evenly presented around the screen with respect to WM locations (Fig. 1a), averaging across relative distractor positions reveals target-related spatial representations because distractor representations are washed out during the averaging procedure. **E** The same data as (**C**, **D**) now aligned to each trial’s distractor position (cyan triangles), and averaged separately for each relative distractor location bin (WM targets are at different locations relative to distractor; orange triangles). **F** Distractor location reconstruction averaged over all relative WM target location bins.

To test our hypothesis that an attended distractor stimulus disrupts neural WM representations, we implemented an inverted encoding model (IEM) of polar angle to visualize and quantify spatial WM representations along an isoeccentric ring of target positions. We trained the encoding model using delay-period data from a single-item memory-guided saccade task collected over 2–3 fMRI sessions independent of the main experimental task. Then, using this fixed encoding model^41,42^, we reconstructed 1D spatial representations from each timepoint of each trial (Fig. 3A, B). Using a fixed encoding model allows us to compare reconstructions between timepoints and task conditions on the same footing, because an identical encoding model is inverted to compute reconstructions across all timepoints and task conditions. Evident even in single trials, the location of the WM target emerged in the reconstructions shortly after the appearance of the stimulus and persisted throughout the entire delay period, while on distractor-present trials the position of the distractor briefly appeared and disappeared in the reconstruction (Fig. 3B).

Testing our hypothesis requires visualizing WM representations independent of distractor representations. To accomplish this, we aligned reconstructions across trials based on the remembered target location, which averages over all relative distractor locations (Fig. 3C, D). Similarly, to visualize the representations of the visual distractor, we aligned the same data based on the distractor location, which averages over all relative target locations (Fig. 3E, F, as in Rademaker et al.^22^. Importantly, because distractors were presented with equal likelihood at one of seven location bins relative to the WM target (Fig. 1A), we were able to independently visualize and assay WM representations and distractor representations on distractor-present trials.

**Fig. 4:**
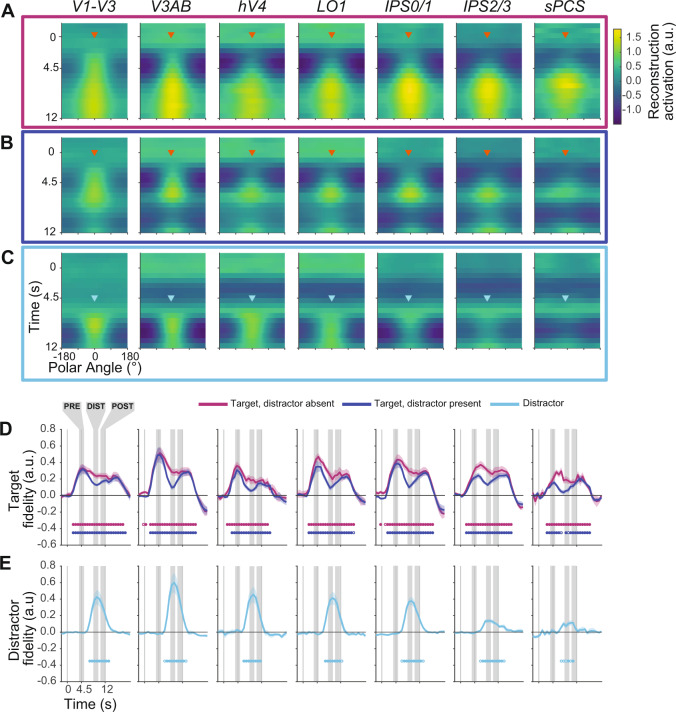
Impact of distraction on the dynamics of WM representations. Average reconstruction of WM target locations on distractor-absent (**A**) and distractor-present trials (**B**) across all participants (*n* = 7). **c** Reconstruction of distractor locations on distractor-present trials, where all trials were aligned to a fixed distractor location. Note that **B** and **C** include the same data, just aligned to different locations (see Fig. 3). Reconstruction strength (arbitrary units, a.u.) is greatest at the aligned location in each instance and represents the polar angle location of the WM target maintained over the entire delay period or the briefly presented distractor. **D**, **E** Fidelity of the neural representation of WM targets (**D**) and distractors (**E**). When activation peaks in the direction of the remembered target (after alignment), fidelity is positive; when there is no consistent activation peak, fidelity is near zero. Target fidelity on distractor-absent trials is robust and statistically significant throughout the delay period in all ROIs. When the distractor is present, fidelity drops, but remains significantly above zero for all ROIs except for one 750 ms TR in sPCS. Distractor fidelity is also statistically significant in all regions and is qualitatively most robust across extrastriate visual cortex (e.g., V3AB). Closed and open circles denote significance of *p* < 0.05, one-sided, FDR corrected and *p* < 0.05, one-sided, FDR uncorrected, respectively (one-sample *t*-test using null distribution derived from shuffled IEM; see Methods section). Error bars ±SEM.

**Fig. 5:**
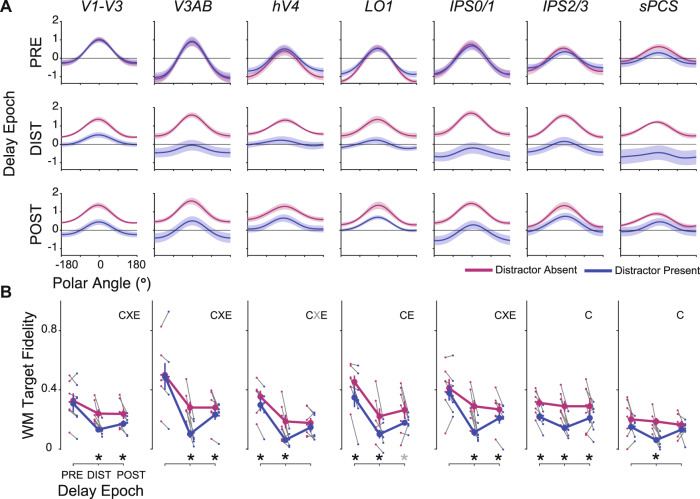
WM representations are transiently disrupted by an attended distractor. **A** Independently trained model-based reconstructions of the WM target locations on distractor-absent (magenta) and distractor-present trials (blue) each averaged over three epochs of the memory delay. The epochs were composed of TRs before the distractor (3.75–5.25 s), during the distractor (8.25–9.75 s), and after the distractor (10.5–12 s), accounting for the hemodynamic delay. Error bars ±SEM. Changes in the baseline (vertical offset) of these reconstructions, which may be due to differences in mean univariate BOLD activation, do not impact fidelity computations (Fig. 5B). Note that during the distractor epoch, the reconstructions of the WM target locations appear weaker on distractor-present compared to distractor-absent trials. In some regions, this effect of the distractor lasts into the post-distractor epoch. **B** Group mean (error bars, ±SEM) fidelity of reconstructed WM targets on distractor-absent (magenta) and distractor-present (blue) trials separately for the pre-distraction, distraction, and post-distraction epochs. Thin gray lines connect mean distractor-absent (small magenta dots) and distractor-present (small blue dots) fidelity for individual participants (*n* = 7) for each delay epoch. The results from 2-way ANOVAs for each ROI (epoch and condition as factors; compared against a shuffled null) are marked by symbols to denote the significant main effects of condition (C), epoch (E), and the interaction between epoch and condition (X). The significant results of paired (two-tailed) *t*-tests between distractor-present and distractor-absent reconstructions per epoch, for each ROI, are marked with asterisks. In both cases, gray symbols denote *p* < 0.05, uncorrected, and black *p* < 0.05, FDR corrected across ROIs within-test. Results for individual ROIs shown in Supplementary Fig. 4, and all *p*-values available in Supplementary Table 4.

Across all participants, the WM target locations were robustly encoded in the modeled population responses in all visual, parietal, and frontal ROIs during distractor-absent trials (Fig. 4A). This demonstrates the robustness of our model, experimental data, and procedures, and replicates previous results^10,16,43^. While some ROIs (e.g., hV4, V3AB, LO1) show lower activation values in the reconstructions at earlier timepoints (see also Fig. 5A), the critical feature of these reconstructions is a greater activation near the WM target location than at other locations. These amplitude differences are expected due to the use of a fixed encoding model (see above), and echo similar differences observed previously^22^.

Turning to the distractor-present trials, the strength of WM representations took a noticeable dip following the distractor presentation (Fig. 4B). This dip, however, was brief and the WM target representation returned shortly after the distractor disappeared, and prior to the response period at the end of the trial. Additionally, by aligning the same data to the distractor location rather than the WM target location (Fig. 4C), one can see strong representations of the distractor locations encoded in the population response of all ROIs.

### Distraction impacts WM representations

Thus far, qualitative examination of WM reconstructions suggests WM target representations are transiently disrupted by an attended distractor stimulus across visual, parietal, and frontal cortex. To quantify and test this hypothesis, we computed a fidelity metric^22,43,44^, which measures the strength of the representation within the reconstruction. Reconstructions of correct WM target locations result in larger positive fidelity values, while poorly matched reconstructions produce low values. Importantly, fidelity can be computed independently for both the WM target and distractor locations because their relative locations were carefully counterbalanced and distributed (i.e., the average distractor location relative to an aligned WM target location has a fidelity of zero). On distractor-absent trials, the fidelity values in all ROIs grew significantly time-locked to the WM target presentation and remained above chance throughout the delay indicating accurate and sustained WM target encoding (Fig. 4D). On distractor-present trials, we observed a phasic increase in fidelity values for the distractor location time-locked to the onset of the distractor in all ROIs (Fig. 4E). Moreover, the fidelity of the WM target representation dropped in all ROIs in the time-points corresponding to the phasic distractor response (Fig. 4D). Nonetheless, even during this dip in target fidelity, the values for the WM target remained significantly greater than zero indicating that the population response still contained information about the WM target location (Fig. 4D, E, one-sided *t*-test at each timepoint compared against a null distribution computed with a model estimated using shuffled trial labels; FDR corrected within each ROI).

### Recovery from the effects of distraction

To further characterize the temporal dynamics of how WM representations are impacted by and recover from distraction, we computed reconstructions and associated fidelity values corresponding to epochs before, during, and after distraction (Fig. 5; see Figs. 1B and 4D, E for epochs). First, to test for differences in WM target fidelity across ROIs, task conditions, and trial epochs, we performed a 3-way repeated-measures ANOVA against a shuffled null distribution with factors of ROI, epoch, and condition (see Methods section: statistical procedures). The 3-way interaction was significant (*p* = 0.041), as well as 2-way interactions of epoch × condition (*p* = 0.001), epoch × ROI (*p* < 0.001), and additionally, main effects of epoch, condition, and ROI (*p* < 0.001; *p* = 0.001; *p* < 0.001). These results suggest that attending a distractor stimulus differentially impacts the WM representation across ROIs and across the delay period. Following up on the 3-way ANOVA and motivated by the dip in fidelity time-locked to the distractor onset (Fig. 4D), we performed a 2-way repeated-measures ANOVA (factors of condition and epoch) within each ROI to formally test if the distractor disrupted the fidelity of the WM target representations. We observed a significant main effect of condition in all ROIs (all *p*’s < 0.037), main effect of epoch in V1-V3, V3AB, hV4, LO1, and IPS0/1 (all *p*’s< 0.014), and interaction in V1-V3, V3AB, and IPS0/1 (all *p*’s < 0.005; tests FDR corrected across ROIs; *p*-values computed against a shuffled null; see Methods section; Fig. 5B).

To unpack these ANOVAs, we performed follow-up *t*-tests within epochs comparing target fidelity on distractor-present and distractor-absent trials. First, WM target representations were significantly disrupted during the distractor epoch in all ROIs (Fig. 5B, DIST: *p’*s < 0.01; FDR corrected across all ROIs for each epoch independently). Second, WM target fidelity remained lower following distraction compared to the same late epoch on distractor-absent trials in V1–V3, V3AB, IPS0/1, and IPS2/3 (Fig. 5B, POST: *p*’s ≤ 0.03). This suggests that the distractor degraded the quality of the WM target representations, which were not completely restored after distraction. Third, immediately following the cue that indicated whether the trial would contain a distractor, WM target fidelity was lower on distractor-present trials even prior to distractor onset in hV4, LO1, and IPS2/3 compared to distractor-absent trials (Fig. 5B, PRE: *p*’s ≤ 0.026). This suggests that when participants know a distractor will appear during a trial, they may be adopting a strategy whereby they encode WM representations in a different format during distractor-present trials than that used for distractor-absent trials (see also Bettencourt and Xu^21^). Next, we test this possibility.

### Representational format of WM does not change during distraction

**Fig. 6:**
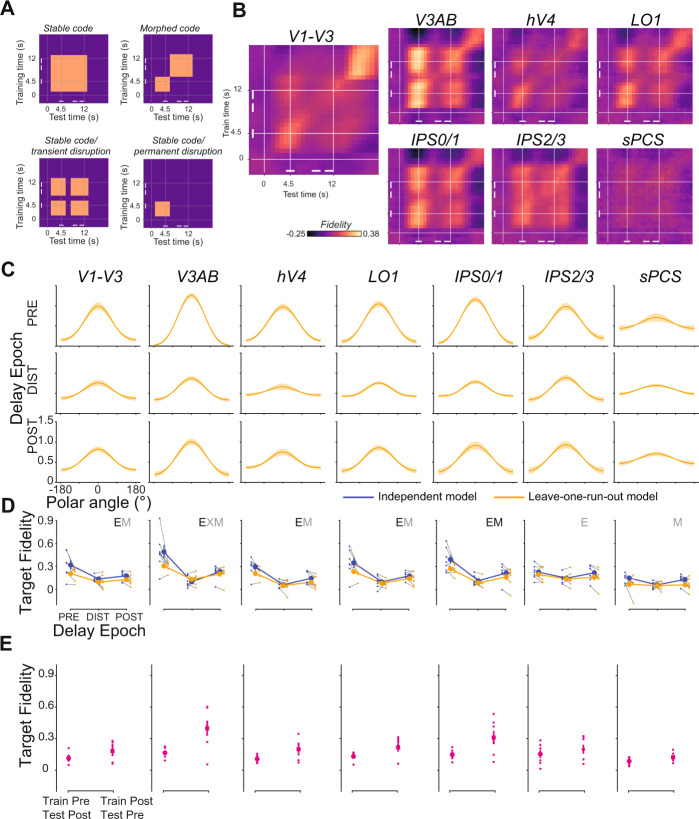
Loss of WM fidelity during distraction cannot be explained by a different coding format. **A** To evaluate the format of WM representations throughout distractor-present trials, we conducted a temporal generalization analysis using distractor-present trials to estimate an IEM (each timepoint in turn) which was used to reconstruct held-out distractor-present trials (each timepoint in turn; leave-one-run-out cross-validation). For each combination of training and testing timepoints, we compute the WM target representation fidelity. Four cartoon examples illustrate predicted results from this analysis under various (non-exhaustive) coding schemes. **B** Fidelity is strong across a large combination of training/testing timepoints during the delay period with no evidence of a transition to a new coding format during or after the distractor. In many ROIs (e.g., V3AB), results are consistent with a transient disruption in WM representation, but no change or morphing in representational format following distraction. White bars indicate epochs used for analyses in **C**, **D**. **C** Model-based reconstructions from a cross-temporal generalization analysis in which training and testing was performed on corresponding epochs of the delay (i.e., train IEM with PRE timepoints, reconstruct using PRE timepoints from trials in held-out run). Rows show reconstructions from each ROI from each epoch (error bars ±SEM). Qualitatively, a substantial dip in WM reconstruction strength is apparent during the DIST epoch, as in Fig. 5A. **D** Comparison of group mean (error bars ±SEM) fidelity during each trial epoch across model estimation procedures. Blue line shows data computed using an independent model (replotted from Fig. 5B); orange line shows data computed using the leave-one-run-out cross-validation procedure. Gray lines connect datapoints from individual participants (*n* = 7). We performed a 2-way repeated-measures ANOVA against a shuffled null for each ROI (factors model and trial epoch). Main effects of model are indicated by M, main effects of epoch are indicated by E, and interactions are indicated by X. Significant tests are shown in black (*p* < 0.05, FDR corrected across ROIs within test); trends are shown in gray (*p* < 0.05, no correction). Error bars ±SEM. No ROIs show a significant interaction between model and epoch (though a trend is seen in V3AB, which is largely driven by stronger WM target representations measured using the independent model). **E** Comparison of off-diagonal training/testing combinations, (group mean, error bars ±SEM, derived from *n* = 7 participants). To determine if models trained and tested on non-matched epochs were able to recover information, we measured fidelity from models trained on PRE and tested on POST, as well as trained on POST and tested on PRE delay epochs. All *p*’s < 0.05 when comparing each average fidelity against a null distribution of fidelity values computed as in Fig. 4. Data from all individual ROIs available in Supplementary Fig. 5; *p*-values for all tests available in Supplementary Table 6.

Previous research^15,21,23^ suggests that participants may proactively insulate WM representations from the deleterious effects of sensory distraction by representing WM features in distinct formats in association cortex compared to visual cortex. Unique representational formats would thus protect at least some WM representations (those in parietal cortex) from sensory interference, while WM representations held in a static or fixed format would be susceptible to sensory interference. To test this hypothesis, we analyzed distractor-present trials using a modified model training/testing procedure. Rather than estimating a model using several independent sessions of independent mapping data, we instead trained the model using distractor-present trials only, using leave-one-run-out cross-validation. Additionally, to evaluate the possibility of a shifting/dynamic code over the delay interval^45,46^, we repeated this procedure for each pair of timepoints during the delay period such that each time point was reconstructed with a model trained at each timepoint^47^. If, during distraction, the dip in WM representation fidelity we observe is explained by a reformatting or recording of information during distraction, this matched model training/testing procedure should show less evidence for distractor interference (Fig. 6A: stable code). Additionally, if reformatting the WM representation effectively insulates it from the disruptive effects of distraction, we would expect to see improved fidelity of the WM representation computed using the leave-one-run-out cross-validation procedure compared to the fidelity of the representation computed using the independently trained model^48^. A non-exhaustive set of other possible results are qualitatively depicted in Fig. 6A. For example, the code may morph following distractor presentation, resulting in a new—but incompatible—WM representation format (Fig. 6A: Morphed code;^34^). Alternatively, if the distractor presentation transiently or permanently disrupts the WM representation, we would observe a brief (or sustained) dip in WM reconstruction fidelity, but no change in its format (Fig. 6A: stable, with transient/permanent disruption).

Using these procedures, WM target fidelity was very stable, with variations in the training or testing time points having little effect on our ability to reconstruct WM representations (Fig. 6B), Additionally, this approach still resulted in apparent loss of WM target information following distraction in visual (V1-V3, V3AB, hV4, LO1) and posterior parietal (IPS0/1) cortex, counter to what would be predicted if the WM representations were transiently transformed into a different format (Fig. 6C, D). These results are therefore incompatible with the hypothesis that WM representations are dynamically recoded into more durable representations as a means to resist the effects of distraction and are most aligned with the stable with transient disruption model depicted in Fig. 6A. Additionally, we did not see evidence for improved WM representation fidelity during the DIST epoch for data reconstructed using the leave-one-run-out model compared to the independent model (Fig. 6D). These data are comparable, since only the model estimation procedure differs between datapoints (the same data in voxel space are transformed by different IEMs).

Next, we quantitatively tested whether the evolution of fidelity of WM representations over the trial differed across model estimation procedures. We computed fidelity for reconstructions generated using a leave-one-run-out cross-validation scheme where a model is trained and tested on matched timepoints (Fig. 6D, orange) and compared these values to those computed using the independent model estimation procedure (same data as in Fig. 5C replotted; blue). First, we performed a 3-way repeated-measures ANOVA (factors of ROI, model estimation procedure, epoch; compared against a shuffled null): there were significant main effects of ROI (*p* < 0.001), model estimation procedure (*p* = 0.01), and epoch (*p* < 0.001), interactions between ROI × epoch (*p* < 0.001) and model estimation procedure × epoch (*p* = 0.029), and a 3-way interaction (*p* = 0.018; all *p*-values available in Supplementary Table 6). Next, we performed 2-way repeated-measures ANOVAs for each ROI (factors of model estimation procedure, epoch; compared against a shuffled null, FDR corrected across ROIs within test). We observed a significant main effect of epoch in V1–V3, V3AB, hV4, LO1, and IPS0/1 (*p* < 0.001), and a main effect of model estimation procedure in IPS0/1 (*p* = 0.004). We did not observe any significant interactions between model estimation procedure and epoch, as would be predicted if WM representations are dynamically insulated from disruption via a distinct coding scheme (all *p*’s>0.079), though a trend for this interaction (*p* < 0.05, no correction for multiple comparisons) was observed in V3AB. Numerically, during the DIST epoch, WM representation fidelity remains lower using the leave-one-run-out cross-validation procedure than using the independent model estimation procedure, which is inconsistent with a protective recoding of WM representations.

We performed an additional analysis to determine whether any reformatting of the WM representation occurred with distraction. We reasoned that, if the WM code is permanently morphed by the distractor, then training the IEM using data from the PRE epoch and testing with the POST epoch (and vice versa) should not recover significant WM representations. Instead, per the stable coding or stable coding with transient disruption model (Fig. 6A), we would expect that WM representations should be recoverable using a model estimated at a different point in time (here, >5 s apart). In both cases and in all ROIs, we were able to decode the target location despite training and testing at epochs before and after the distractor (all *p*’s < 0.05; Fig. 6E and Supplementary Fig. 5c). Altogether, these results suggest the WM representation remains intact in its original format following distraction.

### Distractor-induced offsets in neural representations predict WM errors

Our results thus far indicate that an attended distractor impacts both the quality of WM (decrease in precision and increase in RT; Fig. 1D, E) and the quality of neural WM representations in visual, parietal, and frontal cortex (Figs. 4 and 5). Next, we ask to what extent are changes in WM performance related to distractor-induced perturbations in neural representations? We predict that memory errors (i.e., memory-guided saccade endpoints relative to the true WM target locations) will be attracted towards the location of the distractor stimulus. Moreover, we predict trial-by-trial variability in neural representations of WM targets will correlate with the direction and amplitude of memory errors. To test this prediction, we restricted our analyses to trials in which the target and distractor were near one another (within 12° polar angle), where the angle of attraction towards the distractors would align with the polar angle component of the encoding model (far distractors, instead, would predominantly align with the unmodeled eccentricity component).

**Fig. 7:**
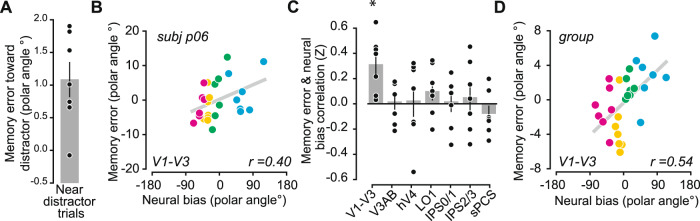
Memory errors correlate with distractor-induced biases in WM representations in visual cortex. **A** On distractor-present trials in which the distractor was presented within 12° polar angle from the WM target, we found an attractive bias such that behavioral WM responses were drawn toward the distractor (positive values indicate errors in the same direction as distractor; two-tailed permutation *t*-test, *p* = 0.006). **B** We quantified the trial-by-trial error of each WM reconstruction based on its circular mean (see Methods section) during the post-distractor epoch on distractor-present trials when the distractor was presented near the WM location. To determine whether behavioral WM responses were impacted by any offsets in these neural WM representations, for each ROI and participant we correlated each trial’s decoded WM representation error with the corresponding behavioral memory error. Example scatterplots shown for one participant and ROI; trend line shows least squares linear fit (all participants and ROIs are plotted in Supplementary Fig. 6). **C** Average (±SEM) neural/behavioral error correlation across participants based on decoded error from each ROI. Behavioral responses significantly correlated with errors in neural representations in V1–V3, but not other ROIs (*p* = 0.005, FDR corrected across ROIs; trial-level permutation test; see Methods section). There was no significant main effect of ROI (*p* = 0.08, permuted 1-way ANOVA). All p-values available in Supplementary Table 8. **D** Additionally, we sorted each trial into quartiles based on each neural bias and subsequently took the mean of neural bias and behavioral memory error within each group of trials per participant. Then, we computed the Pearson correlation coefficient using these binned data (one mean per participant per quartile, 28 data points total). Asterisks denote significant results, p < 0.05, FDR corrected.

We first tested whether memory errors were attracted to the nearby distractor stimulus. We quantified distractor-induced bias as the mean polar angle of memory-guided saccade endpoints flipped and rotated such that the nearby distractor was always clockwise of the WM target location. Indeed, WM errors were significantly biased in the direction of the attended distractor *(*two-tailed *t*-test compared to shuffled null, *p* = 0.006; Fig. 7a). Next, we tested if trial-by-trial biases in the neural representations of WM targets predicted the behavioral memory errors attracted to the distractor locations^32^. To do so, we decoded the WM target location represented by each ROI’s WM reconstructions by taking the circular mean of the reconstruction on each trial during the POST epoch, just prior to the behavioral response (see Methods section). Then, for each participant, we correlated each trial’s decoded WM location with the memory error on the corresponding trial (Fig. 7b; correlations and scatterplots for all individual participants shown in Supplementary Fig. 6). Finally, we aggregated correlation values across participants and compared each ROI’s sample against zero (one-tailed *t*-test against shuffled null). Strikingly, biases in the V1-V3 representations of the WM targets significantly predicted memory errors (*p* = 0.005; corrected for multiple comparisons via FDR), but no such correlations were found in other ROIs (Fig. 7c; all *p*’s > 0.104 and do not survive FDR correction; individual ROI analyses shown in Supplementary Fig. 6), and a shuffled 1-way repeated-measures ANOVA with ROI as the factor was non-significant (*p* = 0.078). To further validate the single-trial, individual participant-level correlations, we computed correlations between binned decoding error and memory error pooled across participants^32,49^. We first binned the decoding errors for each participant into quartiles and computed the mean memory error across each bin. Then, we computed Pearson correlation coefficients at the group level between binned decoding error and memory error, where each participant contributed four data points (one from each quartile bin). Similar to the single-trial correlation, the binned correlation in V1–V3 alone survives permutation testing and FDR correction (Fig. 7d; *p* = 0.007; all other ROIs *p’s* *>* 0.218). These results indicate that WM representations encoded in the population activity of visual cortex are not only susceptible to interference from an attention-demanding distractor task, but that the distractor distorts the neural representation causing systematic errors in memory.

## Discussion

We tested how distraction interferes with working memory (WM) representations to address the debated roles of early visual cortex versus association cortex. Within a widely distributed collection of visual retinotopic maps that encoded spatial WM targets, diverting attention to a salient visual stimulus caused a transient disruption, but not loss, of information about the memorandum. WM representations in parietal cortex were impacted in anticipation of distraction but showed no evidence of undergoing any transformation into a new format. Moreover, distraction caused systematic biases in memory errors, which correlated with trial-by-trial errors in the WM representations encoded in early visual cortex, but not association cortex. Based on these results, we conclude that the neural mechanisms housed in early visual cortex play a central role in WM storage, confirming key tenets of the sensory recruitment model of WM. This conclusion that visual cortex WM representations play a critical role in WM behavior may be surprising as the functions of early visual cortex have historically been limited to visual perception. Cognitive functions like attention clearly modulate neuronal activity in visual cortex measured with fMRI^50,51^ or intracranial electrophysiology in humans^52^ and nonhuman primates^53–55^. The sources that control attention, however, are thought to originate in frontal and parietal association cortex^56^. Moreover, based on electrophysiological studies, the evidence that neurons in visual cortex show persistent activity during memory delays is highly inconsistent (e.g.,^57–59^ recently reviewed in^19,35^. Notably, WM impairments caused by PFC lesions were instrumental in establishing the link between PFC integrity and WM theory^60,61^, but no such evidence can be obtained in the case of visual cortex because visual impairments would confound any conclusions about memory.

The absence of evidence leaves equivocal the role of visual cortex in WM, but studies comparing the effects of distraction have yielded the most damaging evidence against the importance of WM representations in visual cortex. Neural activity in monkey inferotemporal cortex is less robust during memory delays and the selectivity of activity appears to be disrupted by intervening distractors, while PFC representations appear resistant to distraction^4,62^. Similarly, memoranda-specific delay period activity of neurons in the monkey PFC resists the effect of distractors, unlike neurons in posterior parietal cortex^63–66^. The results from these distractor studies of single neurons are widely cited to support the claim that the PFC, rather than posterior cortical areas, is critical for WM storage. In contrast to these results, here we report that WM representations in frontal, parietal, and early visual cortex all survive distraction. The distractor does cause dips in the fidelity of the memory representations, but they are quickly restored to pre-distractor levels throughout the cortex. Indeed, the contents of WM may be protected, not by some specialized control process unique to PFC, but by the widely distributed nature of the WM representations.

More generally, and in contrast to the electrophysiological studies in monkey cortex, a decade of model-based fMRI studies have demonstrated that the patterns of activity in visual cortex, including V1, can be used to decode and reconstruct the contents of WM^5–7,10,16,17,67,68^ recently reviewed in refs. ^9,35^. Moreover, memory-specific activity persists in V1 neurons^58,59^ and V1 voxels^31^ whose receptive fields match the visual field location of WM targets (Fig. 2). Although these results together provide positive evidence in support for the sensory recruitment model of WM^1,12,13^, several aspects of the model remain controversial. One common criticism^18,20,21^ points out that, if visual cortex is maintaining WM information, how can it simultaneously process retinal input? Specifically, it remains unclear if the neural circuitry in early visual cortex can simultaneously maintain WM representations while encoding incoming percepts, like distracting stimuli. Also, if distraction disrupts WM representations encoded in early visual cortex without impacting the quality of WM, then visual cortex’s role in WM might be limited, for instance, to artificial laboratory experiments with blank memory delays.

Much of the recent debate regarding the impact of distraction has specifically involved the relative roles of parietal cortex and visual cortex in WM. Consistent with the previous fMRI studies of distraction^21–23^, visually-engaging or, in our case, attention-demanding distractors have a more reliable adverse impact on behavior and a disruptive effect on WM representations encoded in early visual cortex. Our results differ from those previously reported in several important ways. Previous studies drew conclusions based, in part, on an inability to consistently decode WM contents in visual cortex following distraction^21,23^, or a decrease in decoding performance following distraction along with a decrease in behavioral performance^22^. Here, we generate positive evidence that provides strong support for a critical role for visual cortex in WM^69^. First, we find that visual cortex maintains joint representations of both sustained WM targets and transient attended distractors. These representations were impacted by, but were ultimately robust to, distraction as they were fully restored before the response period (Figs. 3–5). We note that it is possible that WM information was completely lost during distraction, if only for an instant, and our measures were insensitive to such brief distraction. If so, the WM representations were immediately restored. Regardless, even if we had observed a complete loss of WM target information during the distractor period, such absence of evidence cannot be taken as evidence of absence^69^. Second, and most importantly, in our study trial-by-trial distortions in these restored WM representations in visual cortex were positively correlated with errors in memory reports (Fig. 7). The positive relationship between neural representations of WM measured with BOLD fMRI and the quality of WM performance measured later in time is difficult to explain away. In contrast, the studies that have only shown disrupted WM representations during distraction rely on an absence of evidence, which, again, may not be evidence for the absence of a WM representation. Below we discuss the impact of these new findings in the context of the theoretical mechanisms by which early visual cortex and association cortex might support WM.

Previous fMRI studies suggest that WM representations in parietal cortex are resistant to distraction and therefore support the resilience of WM^21–23^. Presumably, parietal cortex stores WM representations in a format distinct from the sensory code used for perception, otherwise the visual input during distraction would interfere with the WM representation. Indeed, Rademaker et al. showed that parietal cortex encodes the remembered orientation, but that the representation can be in a distinct format from that of an attended oriented grating, and that this representation is protected from distracting input. Our results are inconsistent with this explanation. First, we find that distraction does impact WM representations in parietal cortex, if only for a brief period of time (Figs. 4–5). Second, only in early visual cortex do we find that decoding errors predict distractor-induced memory errors (Fig. 7). Third, based on different training/testing procedures over time, we find no evidence that WM target representations are anything but stable codes of the visually presented WM targets (Fig. 6).

Perhaps the type of memorized stimulus matters. Stimulus features that can be condensed or compressed could very well undergo recoding to minimize the demands on memory. For instance, it is unlikely that memory for dot motion is a replay of hundreds of dots moving over time, but instead a single vector representing the compressed bit of task relevant information. Therefore, the absence of motion-related activity during a memory delay in area MT is unsurprising^57,70^. A spatial location, however, may not be compressible and therefore does not necessitate any transformations. Even if WM representations in parietal cortex are more distractor-resistant, it remains unclear why. The WM code might be less sensory in nature making it in turn less like incoming percepts, or control processes might actively protect their contents^71^. Interestingly, in our data, we find that WM representations in parietal cortex were affected by the mere anticipation of distraction (Fig. 5). Following the cue indicating the trial would contain distraction, the fidelity of the WM representation was lower compared to trials with no distraction. As discussed above, we ruled out the possibility that the WM representations dynamically morphed over time (Fig. 6). We hypothesize that storage of the target position and covert attention needed to rapidly detect the distractor share a common mechanism akin to the control of spatial attention^8^. The drop in WM fidelity we observed in parietal cortex may stem from interference with the control of attention, rather than the storage of WM^72–75^. Regardless, WM representations in parietal cortex had no bearing on the changes in WM behavior induced by the distractor. A strong test of the importance of a neural WM representation lies in its ability to predict WM behavior^20,69^. In this study, we measured neural and behavioral biases on each trial and found a strong positive correlation between the two in visual cortex alone. These results at the trial-level provide positive evidence for a critical role of visual cortex in WM, and extend previous demonstrations linking the quality of WM decoding to individual differences in WM performance^14,17,48,68,76^ and differences averaged over conditions^14,43^. Note that the range of behavioral WM errors were much smaller than the range of decoded errors. Indeed, similar mismatches between behavioral error and decoded error have been reported both at the single-trial level^32,49^ and when trials are aggregated^23^. The cause of this mismatch remains unknown. One possible cause is unexplained measurement noise in the BOLD signal, which is not a direct readout of local neural activity. Additionally, the distributed nature of WM representations may dampen the impact on behavior that biases in the representation in one area may have. Finally, while in principle the visual representation of the distractor stimulus itself could leak into the reconstructions computed at the end of the delay period, this could not easily explain our results. We focused our trial-wise correlation analyses on trials with distractor stimuli presented nearby the WM location. Such small differences could not induce neural biases that were much larger in scale, and thus, our results are unlikely to only reflect lingering sensory-evoked activation.

There are several properties that may constrain the mechanisms by which visual cortex supports WM storage. Theoretically, microcircuits that support WM through memory-specific persistent activity are supported by excitatory recurrent connections^77,78^. The slow kinetics of NMDA receptor mediated currents in PFC support persistent activity^79^, while theoretical models suggest that the faster decay rates, like those in V1^80^, would limit persistent activity^81^. In general, intrinsic neural dynamics slow as one moves from visual, to parietal, to frontal cortex^82^. Interestingly, this trend can be seen in our average BOLD time courses, where delay period activity increased systematically along the dorsal visual stream, consistent with our previous reports^8,31^. Beyond the temporal domain, relative differences in anatomical properties also suggest association cortex may have advantages over visual cortex in its capacity for WM storage. The density of NMDA receptors are less expressed in V1 than PFC^80^. Pyramidal neurons in association cortex, compared to visual cortex, have larger and more complex dendritic branching with a greater number of spines^83^, and have more extensive horizontal collaterals in Layers II and III^84^. Together, these anatomical features may better equip association cortex with an increased capacity to integrate inputs, including the excitatory connections theorized to form positive feedback loops to sustain WM representations^85^.

Then what role does early visual cortex play in the maintenance of WM representations? First, consider that the differences between visual cortex and association cortex in temporal dynamics and anatomy are all relative. In fact, when the recurrent network theory of WM was first proposed^85^, several of the hypothesized features of PFC circuitry were unknown and were simply extrapolated from V1 (e.g., recurrence supported by horizontal connectivity between similarly tuned neurons^86^. In theory, the same type of recurrent network could sustain WM representations in V1 as long as the rate of excitatory feedback inputs are greater than the rate of decay. Perhaps a critical source of the feedback is not local but originates from frontal or parietal cortex. Such a mechanism is central to the sensory recruitment theory of WM, where top-down attention signals are proposed to target and boost sensory neurons to prevent memory decay^1^. Second, the factors that presumably make visual cortex less than ideally suited for WM storage do not preclude it from being a necessary node in a larger WM network. The great precision of our visual WM likely depends on interactions between control mechanisms stemming from the association cortices and the precise encoding mechanisms in early visual cortex, and not separate systems specialized for perception and memory. Beyond WM, such concepts are supported by a growing appreciation of the critical role of visual cortex in reinstating visual percepts retrieved from episodic memory^87–91^ and imagery recalled from semantic memory^92–94^.

With these ideas in mind, we designed our distractor task to not only inject noise into the population-based representation through bottom-up visual stimulation but to also interfere with the top-down signals that might be necessary to sustain WM representations in visual cortex. We found that nearby distractors had an attractive pull on memory, biasing memory errors towards the distractor, similar to previous studies^24–28^. Critically, when the distractor was near the WM target, fluctuations in trial-by-trial neural decoding errors in early visual cortex, but not association cortex, predicted WM errors (Fig. 7). Previous studies have only reported that WM decoding errors in visual cortex predict whether distractors were clockwise or counterclockwise relative to the memoranda^23^ and that individual differences in WM performance can be predicted by average decoding accuracy of delay period activity in visual cortex^10,14,17,48,68,76^. Our results are consistent with bump attractor models of WM that assume WM representations are self-sustained by the collective response of populations of neurons whose tuning varies along a stimulus dimension^77,81,95^. Most relevant, these models predict that small random drifts in the bumps of activity cause the seemingly random inaccuracies in memory. Evidence for this hypothesis exists, as clockwise or counterclockwise biases in population estimates of delay activity in macaque PFC neurons predict small angular errors in memory-guided saccades^33^. Using model-based fMRI in humans, we also find direct evidence to support this hypothesis but in early visual cortex, where angular decoding errors in V1–V3 predicted memory-guided saccade errors, but those in parietal or frontal cortex did not. This coupling strongly suggests that the overt report of one’s memory depends on the read-out of the population’s encoded representation in visual cortex. Ultimately, the question of whether early visual cortex is essential for visual WM, wherever one draws that line, is less relevant than trying to understand the mechanisms by which visual cortex contributes to WM.

## Methods

### Participants

Seven neurologically healthy volunteers (3 female; 25–50 years old) with normal or corrected-to-normal vision participated in this study after giving written informed consent, using procedures approved by New York University IRB (protocol IRB-FY2017-1024). Each participant completed 1–2 sessions of retinotopic mapping and anatomical scans (~2 h), between 2 and 3 independent mapping sessions for model training (~1.5 h each), and two experimental sessions (~1.5 h each). Sample size was chosen based on similar sample sizes recruited for similar studies employing deep, multi-session imaging per participant^22^, *n* = 6;^43^, *n* = 6).

### Stimulus

Stimuli were generated via PsychToolBox in Matlab 2018b on a PC and presented via a contrast linearized ViewPixx ProPixx projector, with resolution 1280×1024 and refresh rate 120 Hz. Participants viewed the stimuli through a mirror attached to the head coil at a viewing distance of 63 cm. The projected image spanned 36.3 cm height, resulting in a maximum field of view of 32.14° visual angle.

### Experimental task

Participants performed a modified version of the memory-guided saccade task^96^ (Fig. 1A, B). All stimuli were presented within a 15° radius circular gray aperture on a black background. Throughout the whole experiment, a 0.075° radius light gray fixation point was presented in the center of the aperture. We tested two conditions, randomly interleaved: distractor-absent trials required participants to precisely remember a target location over an extended delay interval, and distractor-present trials required participants to additionally perform a visual motion discrimination task based on a peripheral visual stimulus. Each trial began with a pre-cue (1000 ms; 0.55° circle centered at the fixation point) reliably informing participants whether a distractor would (cyan) or would not (magenta) be presented during the delay period. This controlled for possible impacts of distractor predictability on cognitive control of WM representations^21,22^. Next, a light gray target dot (0.65° diameter) was presented at a randomly chosen position along an invisible ring 12° around fixation for 500 ms. Participants precisely remembered the target location over the subsequent 12 s delay interval. On distractor-absent trials, no further stimuli appeared during the delay period. On distractor-present trials, 4500 ms into the delay period a distracting stimulus appeared at one of seven positions relative to the WM target position around the invisible 12° eccentricity ring for 1000 ms. On each distractor-present trial, distractor positions were randomly chosen (counterbalanced within a run) and were further jittered by ±12° polar angle. The distractor stimulus was a random dot kinematogram (RDK) containing equal numbers of black and white dots (100% contrast; 17 dots/deg^2^, 0.075° dot radius, 1° radius of dot patch; 0.1 s dot lifetime), with a subset of dots rotating either clockwise or counterclockwise about the center of the dot patch (~100° polar angle/s). Participants responded within 2.5 s of the distracting stimulus onset whether the coherently moving dots rotated clockwise (right button) or counterclockwise (left button). The coherence of the dot patch was fixed within a run, and was adjusted between runs based on behavioral accuracy to achieve ~75% performance (mean = 73%, SEM = 6.74%). Non-coherent dots each moved in a random direction. The remainder of the delay (6500 ms) was identical to the distractor-absent trials.

On all trials, at the end of the full 12 s delay period, the fixation point was replaced with a filled light gray circle, cueing participants to make a saccade towards the remembered position. After 800 ms, the target dot was re-presented for 1000 ms, which participants were instructed to fixate. Each trial ended with a 7–13 s ITI (in 750 ms steps), randomly chosen. In addition, on distractor-present trials, participants received feedback on their discrimination performance at the beginning of the ITI (red or green circle at fixation for correct or incorrect response, respectively). Across two sessions, participants performed between 25 and 36 runs (12–18 runs per session, 279 s per run), where each run consisted of seven distractor-present and three distractor-absent trials, randomly ordered. This resulted in between 250 and 360 total experimental trials per participant.

### Independent model estimation task

We acquired a separate, independent dataset used only to estimate the inverted encoding model for spatial position. Participants performed a simple memory-guided saccade task over a 12 s delay using a nearly identical stimulus display, but with no distractor stimulus. Within each 16-trial run, target locations were chosen from a discrete set of positions spaced evenly around an invisible ring (12° eccentricity), with the set of positions staggered every other run, for 32 unique positions overall. No pre-cue was presented at the beginning of each trial. Participants performed between 20 and 31 runs, across 2–3 separate scanning sessions, where each run was composed of 16 trials and lasted 396 s.

### Retinotopic mapping task

To identify regions of interest (ROIs) for all reported analyses, we acquired retinotopic mapping data based on the population receptive field (pRF) method, similar to refs. ^36,97^. During each retinotopy run, subjects completed a difficult discrimination task within bars that swept across 26.4° of the visual field in twelve 2.6 s steps. Bar widths and sweep directions were pseudo-randomly chosen from three different widths (2.5°, 5.0°, and 7.5°) and four directions (left-to-right, right-to-left, bottom-to-top, top-to-bottom), respectively. Each bar was split into three equally sized rectangular patches along its long axis. Each of the three patches contained an RDK moving in one of the two possible directions perpendicular to the bar’s swipe direction (parallel to the long axis). During each step of the swipe, the RDK direction in one of the peripheral patches matched the RDK direction in the central patch. Subjects were instructed to report, via a button press, which peripheral patch matched the central patch’s RDK direction, at each step of the swipe. After each report, participants received accuracy feedback (red or green fixation point), and a three-down/one-up staircase was implemented to maintain task difficulty ~80%. The coherence of the RDK in two peripheral patches was always 50% while a variable RDK coherence was used in the central patch to adjust the difficulty of the task. Stimulus presentation code is available at github.com/clayspacelab/vRF_stim.

### fMRI acquisition

All functional MRI images were acquired at the NYU Center for Brain Imaging 3T Siemens Prisma Scanner. fMRI scans for experimental, model estimation, and retinotopic mapping were acquired using the CMRR MultiBand Accelerated EPI Pulse Sequences (Release R015a)^98–100^. All functional and anatomical images were acquired with the Siemens 64 channel head/neck coil.

### Experimental and model estimation scans

For the experimental and model estimation scans, BOLD contrast images were acquired using a Multiband (MB) 2D GE-EPI with MB factor of 4, 44 2.5-mm interleaved slices with no gap, isotropic voxel size 2.5 mm and TE/TR: 30/750 ms. We measured field inhomogeneities by acquiring spin-echo images with normal and reversed phase encoding (3 volumes each), using a 2D SE-EPI with readout matching that of the GE-EPI and same number of slices, no slice acceleration, TE/TR: 45.6/3537 ms.

### Retinotopic mapping scans

For the retinotopic mapping scans, BOLD contrast images were acquired using a Multiband (MB) 2D GE-EPI with MB factor of 4, 56 2 mm interleaved slices with no gap, isotropic voxel size 2 mm and TE/TR: 42/1300 ms. Distortion mapping scans were acquired with normal and reversed phase encoding, using a 2D SE-EPI with readout matching that of the GE-EPI and same number of slices, no slice acceleration, TE/TR: 71.8/6690 ms.

### Anatomical and bias images

T1- and T2-weighted images were acquired using the Siemens product MPRAGE and Turbo Spin-Echo sequences (both 3D) with 0.8 mm isotropic voxels, 256 × 240 mm slice FOV, and TE/TR of 2.24/2400 ms (T1w) and 564/3200 ms (T2w). We collected 192 and 224 slices for the T1w and T2w images, respectively. We acquired between two and five T1 images, which were aligned and averaged to improve signal-to-noise ratio. In addition, to correct functional images for inhomogeneities in the receive coil sensitivity and improve the motion correction and coregistration process, we collected two fast 3D GRE sagittal images (resolution: 2 mm isotropic, FoV: 256 × 256 × 176 mm; TE/TR: 1.03/250 ms), one with the body coil and the other with the 64 ch head/neck coil.

### fMRI preprocessing

We used all intensity-normalized high-resolution anatomical scans (for each participant, 2–5 T1 images and 1 T2 image) as input to the ‘hi-res’ mode of Freesurfer’s recon-all script (version 6.0) to identify pial and white matter surfaces. We edited these surfaces by hand using Freeview as necessary and converted surfaces to SUMA format. The processed anatomical image for each participant acted as the alignment target for all functional datasets. Our aim for functional preprocessing was to put functional data from each run into the same functional space at the same voxel size acquired during the task sessions (2.5 mm isovoxel), account for run- and session-specific distortions, incur minimal volume-wise smoothing by minimizing spatial transformations, and apply a marginal amount of smoothing along the direction orthogonal to the cortical surface. This allowed us to optimize SNR and minimize smoothing, ensuring ROI data remains as near as possible to its original dimensionality. Moreover, because the distortion field can depend on the exact position of the head within the main field, we divided functional sessions into 3–5 ‘mini-sessions’ consisting of 1–4 task runs split by a pair of spin-echo images acquired in opposite phase encoding directions, used for anatomical registration and computing distortion fields for distortion correction. We applied all preprocessing steps described below to each mini-session independently, and inspected motion correction, coregistration and distortion correction to ensure the procedures worked as intended. Preprocessing was performed using a combination of scripts generated with AFNI’s afni_proc.py and custom scripts implementing AFNI functions (version 17.3.09, pre-compiled Ubuntu 16 64-bit distribution). We performed all analyses on a LINUX workstation running Ubuntu v16.04.1 using 8 cores for most OpenMP accelerated functions.

First, we corrected functional images for intensity inhomogeneity induced by the high-density receive coil by dividing all images by a smoothed bias field (15 mm FWHM), computed as the ratio of signal in the receive field image acquired using the head coil to that acquired using the in-bore ‘body’ coil. To improve coregistration of functional data to the target T1 anatomical image, we used distortion-corrected and averaged spin-echo images (which were used to compute distortion fields restricted to the phase-encode direction) to compute transformation matrices between functional and anatomical images. Then, we used the rigorous distortion-correction procedure implemented in afni_proc.py to undistort and motion-correct functional images. Briefly, this procedure involved first distortion-correcting all images in each run using the distortion field computed from the spin-echo image pair, then computing motion-correction parameters (6-parameter affine transform) using these unwarped images. Next, we used the distortion field, motion correction transforms for each volume, and the functional-to-anatomical coregistration simultaneously to render functional data from native acquisition space into unwarped, motion corrected, and coregistered anatomical space for each participant at the same voxel size as data acquisition in a single transformation and resampling step. For retinotopic mapping data, this was a 2 mm isovoxel grid; and for task data, this was 2.5 mm isovoxel grid. For both task and retinotopy data, we projected this volume-space data onto the reconstructed cortical surface. For retinotopy data, we made a smoothed version of the data by smoothing on the cortical surface (5 mm FWHM). We then projected surface data (for task data, only the ‘raw’ data; for retinotopy data, the raw and smoothed data) back into volume space for all analyses. For unsmoothed data, this results in a small amount of smoothing for each voxel along a vector orthogonal to the surface in volume space.

To compute pRF properties (see below) in the same voxel grid as task data, we projected retinotopy time series data onto the surface from its native space (2 mm iso), then from the surface to volume space at the task voxel resolution (2.5 mm iso). This ensured that variance explained estimates faithfully reflect goodness of fit and are not impacted by smoothing incurred from transforming fit parameter values between different voxel grids. We linearly detrended activation values from each voxel from each run and converted signal to percent signal change by dividing by the mean over the entire run. For multivariate analyses on task data, we subsequently Z-scored each voxel across all volumes for each run independently.

### Retinotopic mapping and ROI definition

We averaged time series from each voxel across all retinotopy runs (9-12 per participant) in volume space and fit a pRF model for each voxel using a GPU-accelerated extension of vistasoft (github.com/clayspacelab/vistasoft). We fit a compressive spatial summation isotropic Gaussian model^101^;^36^ as implemented in mrVista (see^36^ for detailed description of the model). We created a high-resolution stimulus mask (270 × 270 pixels) to ensure similar predicted response within each bar size across all visual field positions (to mitigate the effects of aliasing with a lower-resolution stimulus mask grid), and began with an initial high-density grid search, followed by subsequent nonlinear optimization. Note that, in these analyses, because we conduct a grid search on all voxels independently, there is no smoothing of parameter estimates applied after this step before nonlinear optimization. For all analyses described below, we used best-fit pRF parameters from this nonlinear optimization step.

After estimating pRF parameters for every voxel in the brain, ROIs were delineated by projecting pRF best-fit polar angle and eccentricity parameters with variance explained ≥10% onto each participant’s inflated surfaces via AFNI and SUMA. ROIs were drawn on the surface based on established criteria for polar angle reversals and foveal representations^36^;^38^;^102^;^103^;^104^. Finally, ROIs were projected back into volume space to select voxels for analysis. In this report we consider data from V1, V2, V3, V3AB, hV4, LO1, IPS0, IPS1, IPS2, IPS3, and sPCS, which are all retinotopic regions described in previous reports examining the impact of visual distractors on WM representations^21–23^. Only voxels with ≥10% variance explained in pRF model fits were included in subsequent fMRI analyses. Additionally, we group ROIs V1-V3, IPS0-1, and IPS2-3 by concatenating voxels before multivariate analyses for results presented in Figs. 3–7 because they belong to clustered defined by overlapping foveal representations^38^, and for consistency with a prior report^23^; see Supplementary Tables and Figures for analyses reported for each ROI individually, including the size and eccentricity relationship between the pRF parameters in Supplementary Fig. 2C and number of voxels in Supplementary Table 1.

### Oculomotor processing

Monocular eye-tracking data were recorded using an MR-compatible Eyelink 1000 infrared eye tracker (SR Research). Eye position (X,Y) and pupil size were recorded at 500 Hz. Prior to the beginning of the experiment, eye position was calibrated with a 13-, or 9-point calibration. Eye data were transformed from raw pixel screen coordinates into degrees of visual angle utilizing the freely available iEye toolbox (github.com/clayspacelab/iEye_ts). First, the data was rescaled given known screen resolution and viewing distance. Next, the data was inspected for extreme values and blinks, which were removed. It was then smoothed with a Gaussian kernel (5 ms SD). Saccades were identified by identifying periods with velocity in excess of 30°/s for at least 7.5 ms and resulting in at least a 0.25° amplitude gaze shift. Then, the data for an entire trial was drift corrected by taking the mean over known epochs when the participant is fixating and subtracting that value from the entire trial. Finally, the data is recalibrated to the target position on a run-wise basis by fitting a 3rd order polynomial to X and Y eye positions independently to best approximate the true target location on each trial. On a given trial, if the initial saccade did not meet the following additional criteria, that trial was also excluded from behavioral analysis, and from correlations with neural data (Fig. 7): less than a total duration of 150 ms, at least 5° in amplitude, and within at least 5° error from the target location. Trials could additionally be removed from analysis if the participant exhibited a fixation break of at least 2.5° during the WM delay or did not make a saccade within the specific response epoch.

### Oculomotor analysis

Once saccades were preprocessed, we used the last endpoint of the memory-guided saccade before the reappearance of the target, after any corrective saccades, as a measure of the participant’s behavioral WM report in our analyses. For all analyses, we realigned all visual field coordinates of both memory-guided saccade and distractor locations with reference to the saccade target location. We subtracted the polar angle of the memory target from the polar angle of the memory-guided saccade while keeping the radius of each location untouched (to preserve the eccentricity of the reported position). For our precision analysis, we calculated each participant’s saccade standard deviation (SD) by computing the across-trial standard deviations of saccadic polar angle. We calculated reaction times by taking the time from the onset of the response cue to the onset of the initial saccade.

### fMRI: univariate analysis

To assess the mean response across spatially selective voxels subtending relevant locations (target/distractor) in all ROIs, we computed an event-related average of measured BOLD response for each condition separately. After extracting *Z*-scored BOLD signal (see Preprocessing) from each voxel, we sorted voxels on each trial according to their best-fit pRF parameters and the known location(s) of the target and/or distractor. RF-in responses (corresponding to voxels tuned nearby the relevant location) were determined by selecting voxels with ≥10% variance explained, eccentricity between 2° and 15°, and polar angle difference between the WM target (or distractor) and pRF center of each voxel ≤15°. RF-out responses were determined by selecting voxels with ≥10% variance explained, eccentricity between 2° and 15°, and a polar angle difference between the WM target (or distractor) and pRF center of each voxel ≥165°. We averaged responses across such selected voxels within each ROI, then across all trials within a condition (Fig. 2, all ROIs in Fig. S2). Additionally, we removed the baseline response measured between −2.25 and 0 s relative to delay onset. In Supplementary Table 1c, we report the number of voxels averaged over participants, as well as additional requisite RF parameters, for a given ROI.

### fMRI: multivariate inverted encoding model

To reconstruct the representation of visual polar angle carried by the neural population activity of each ROI at any given time, we implemented an inverted encoding model (IEM)^105,106^ which maps between a modeled encoding space and the measured response across a set of fMRI voxels using a simplified set of information channels (or basis functions) each with a preferred stimulus value. The model assumes that the activation of each voxel in response to a stimulus can be described as a simplified encoding model built via a linear combination of all modeled channel responses to that stimulus. Based on this assumption, the activation of these modeled channels most likely to give rise to an observed activation pattern can be estimated using the inverse of the encoding model. Thus, we can map one space to the other by estimating the strength of the links (i.e., regression weights) between all voxels in a given region and all of the modeled information channels. Note that this analysis framework does not, and cannot, infer ‘tuning’ properties of neurons within the regions analyzed^107^. Instead, it recovers region-level representations of a feature space (here, spatial position parameterized by polar angle). See Sprague et al.^106^ and Sprague et al.^108^ for a detailed discussion.

To build an IEM for each ROI, we first obtained an independent dataset which we used to calculate the regression weights that describe the encoding model for each voxel (one weight for each channel for each voxel). Model estimation data was always the average delay-period activation between 5.25 and 12 s following delay onset (average over 9 TRs, each 750 ms). While these regression weights enable us to map the stimulus space to the voxel space, we used inverted weights to estimate each channel’s response and used them to reconstruct the stimulus space given the population activity pattern at each timepoint. Through this method, we were able to reconstruct the spatial representation of the memory target and the distractor location from the neural population activity during WM. The linear mapping between the voxel space and the stimulus space is defined as:

1 $$B=CW$$

Where, *B* is a matrix (*n* trials × *m* voxels) consisting of the activity of all voxels in a given ROI across trials, *C* is a matrix (*n* trials × *k* channels) containing the response of all channels across the same trials, and *W* is a matrix (*k* channels × *m* voxels) of regression weights describing how much each modeled channel contributes to the BOLD signal measured in each voxel. As the stimuli always appeared along a fixed annulus of 12° in this experiment, we modeled the information channels as 1-D smooth tuned filters centered at 8 uniformly distributed polar angles ($\psi$) around the 360° polar space (Fig. 2):

2 $${f}_{i}(\theta )=0.5+0.5\times {\cos }(180\times \frac{\theta -{\psi }_{i}}{2s})^{8}\,{{{{{{\mathrm{for}}}}}}}\,{{|}}\theta -{\psi }_{i}{{|}} \, < \, s{{;}}\,0\,{{{{{{\mathrm{otherwise}}}}}}}$$

We used a size constant (s) of 180°. To calculate each channel output from the population activity in response to a given stimulus, we first need to calculate the regression weights (*W*) in (Eq. 1). We can do this using measured activation patterns (*B*_trn_) and corresponding predicted channel responses (*C*_trn_) for model estimation data:

3 $${\hat{W}}=({C}_{{{{{\mathrm{trn}}}}}}^{T}{C}_{{{{{\mathrm{trn}}}}}})^{-1}{C}_{{{{{\mathrm{trn}}}}}}^{T}{B}_{{{{{\mathrm{trn}}}}}}$$

Where *B*_trn_ is a matrix of BOLD activity in response to our training stimuli (trials × voxels) and *C*_trn_ is a matrix containing the corresponding predicted channel outputs calculated from (Eq. 2), given the training stimulus locations (polar angle). For the IEM, we used these regression weights calculated from an independently collected training set to estimate channel outputs from the measured BOLD signal at any given epoch in our WM task. We estimated these channels outputs as:

4 $${C}_{{{{{\mathrm{tst}}}}}}={B}_{{{{{\mathrm{tst}}}}}}{\hat{W}}^{T}({\hat{W}{\hat{W}}^{T}})^{-1}$$

Where *B*_tst_ contains the measured BOLD signal on each time point of experimental trials, $\hat{W}$ contains the regression weights (estimated using an independent dataset, Eq. 3), and *C*_tst_ is the estimated channel responses.

Finally, to reconstruct the stimulus space, given a set of computed channel responses (per timepoint, epoch, or trial), we calculate the sum of all channel sensitivity profiles, each weighted by its corresponding estimated channel response. For visualization, we align each trial based on the known target location (Figs. 2C, D and 3A, B) or the known distractor location (Figs. 2E, F and 3C) by circularly shifting the reconstruction such that the aligned position is denoted as 0°. Note that for distractor-present trials, the reconstruction necessarily contains both a representation of the distractor and a representation of the target location. But, because these were randomized with respect to one another, aligning to one results in the other representation ‘averaging out’ (Fig. 2). This procedure differs from previous reports which iteratively shift the basis set (e.g., refs. ^109–111^ or compute a correlation between predicted and reconstructed channel responses for each possible feature value^23,105^, but is consistent with previous procedures applied to 2D spatial IEMs^10,43,112^.

### Model training

For Figs. 3–5, we estimated the IEM using delay-period activation (5.25–12 s) measured from an independent mapping dataset in which participants performed a single-item MGS task (see above). Additionally, in Fig. 6, we tested whether a model estimated using distractor-present trials contains WM information in a neural format different from that used in the independent mapping dataset. We performed a leave-one-run-out cross-validation procedure whereby all distractor-present trials from all runs but one (concatenated across sessions) are used to estimate the IEM, and this IEM is used to reconstruct WM representations from the held-out run. We repeat this procedure over all runs, until each distractor-present trial has served as a ‘test’ trial.

Moreover, to establish whether the format of WM representations transforms over the delay period following disruption by a distractor (e.g., refs. ^34,45^, we performed this leave-one-run-out analysis for each pair of training/testing timepoints during the trial (Fig. 6A), and for each pair of trial epochs (pre-distractor, distractor, post-distractor; Fig. 6B–D). Note that, in this analysis, we are not modeling any effect of the distractor (that is, we’re estimating the encoding model using predicted channel responses computed based only on the WM target position). Because we do not have a strong understanding of the format of distractor representations, nor how these interact with WM representations, we cannot build these features into our model. Regardless, reconstructions computed using this approach can characterize activation patterns associated with WM targets, even if the estimated model remains imperfect (for a similar approach, see Iamshchinina et al.^48^ analyses employing a leave-one-run-out procedure within each task condition).

### Fidelity

To quantify the presence of information about a remembered or viewed stimulus location in IEM-based reconstructions, we computed a model-free index of ‘fidelity’^22,43,44^. Conceptually, this metric measures whether the reconstruction, on average, ‘points’ in the correct direction, along with how ‘strong’ the representation is. Accordingly, we compute the vector mean of the reconstruction in polar coordinates. When reconstructions are rotated and aligned to 0°, projecting this vector mean on the horizontal axis captures energy in the reconstruction consistent with the aligned location. Each reconstruction r(*θ*), where *θ* is the polar angle of each point and r(*θ*), is the reconstruction activation) when plotted as a polar plot, was projected along the x-axis (reconstructions were rotated such that the target was presented at 0°):

5 $$F={{{{{\mathrm{mean}}}}}}(r(\theta )\times {{{{{\mathrm{cos}}}}}}(\theta ))$$

If *F* is reliably greater than zero, this quantitatively demonstrates that the net activation over the entire reconstruction carries information above chance about the target position. This measure is independent of baseline activation level in the reconstruction, as the mean of *r(θ)* is removed by averaging over the full circle. We computed fidelity for each timepoint of each trial in the experiment after aligning reconstructions to the target position, and, on distractor-present trials, the distractor position. Because the non-aligned stimulus type (target or distractor) was located at a random and symmetric position with respect to the aligned stimulus type (Fig. 1A), we could independently assay information about both stimulus types on the same set of trials (see also Rademaker et al.^22^).

### Quantifying decoded position

We also used single-trial reconstructions on trials with distractor locations nearby the WM target location to decode the position (polar angle) represented by the population activation pattern. We defined the decoded position (*WM*_est_) as the circular mean of the reconstruction, which we computed by summing over unit vectors pointing in each direction weighted by the reconstruction activation in that direction, then taking the inverse tangent of the resulting vector:

6 $$W{M}_{{{{{{{\mathrm{est}}}}}}}}={{{{{{\mathrm{ta}}}}}}}{{{{{{\mathrm{n}}}}}}}^{-1}\left(\frac{\varSigma r(\theta ){\sin }(\theta )}{\varSigma r(\theta ){\cos }(\theta )}\right)$$

### Statistical procedures

To test whether behavioral performance was impacted by distractor presence or absence, we performed one-way repeated-measures ANOVAs on memory error and saccadic reaction times. Memory error was defined as the standard deviation of target-aligned saccadic polar angle (degrees). Reaction time was defined as time at which an initial ballistic saccade was made in the response period, as measured from the onset of the response period (milliseconds).

To quantify how distraction impacted univariate fMRI responses (Fig. 2 and Supplementary Fig. 2), we first conducted a 2-way ANOVA using ROI and RF condition (in vs. out) on average BOLD delay-period (3.75–12 s) responses from voxels selected by their proximity to (within 15° polar angle; RFin) or separation from (at least 165° polar angle from) the WM target (Fig. 2A, B and Supplementary Fig. 2A, B) or distractor (Fig. 2C and Supplementary Fig. 2C). F-scores computed for each main effect were compared against a distribution of data shuffled within-participant 1000x. *P*-values were determined by the proportion of shuffled F-scores per main effect greater than or equal to the F-score computed using the intact data. We performed follow-up 1-way ANOVAs within each ROI, using RF (in vs. out) as the main effect, and F-scores were computed using the same within-participant shuffling procedure. FDR corrections were performed across ROIs.

To demonstrate the presence of information about the target/distractor location in an IEM-based reconstruction, we computed *t*-statistics on each fidelity timepoint (Fig. 4D-E; 6E) by first creating an empirical null *t*-distribution from shuffling trial labels of the training dataset within each participant 1000x. The sample of fidelity values computed using an intact model at each timepoint was then compared against the T distribution computed using shuffled data. We compared the T-score computed using intact trial labels to this null distribution and defined the *p*-value as the proportion of null T values equal to or exceeding the actual value (one-tailed; Rademaker et al.^22^). We corrected for multiple comparisons using the false discovery rate across timepoints within each ROI and condition.

To quantify WM representation fidelity (Fig. 5) in each ROI during each trial epoch, we first conducted a 3-way repeated-measures ANOVA (ROI × distractor condition × delay epoch) on average BOLD responses across voxels within each ROI and trials within each condition. F-scores computed for each main effect, 2-way, and 3-way interaction were compared against a distribution of each comparison computed using data shuffled within each participant over 1000 iterations. P-values reflect the proportion of null F-scores within each comparison greater than or equal to the F-score computed using intact data. The minimum *p*-value achievable with this procedure is 0.001. We conducted follow-up 2-way repeated-measures ANOVAs for each ROI (distractor condition × delay epoch) using the same shuffling procedure within each ROI. *P*-values were corrected for multiple comparisons across all ROIs within each comparison (separately for each main effect and interaction; FDR). We use a similar analysis to compare between model training procedures (train with independent model vs train with leave-one-run-out cross-validation) to test the main effect of model training procedure, epoch, and their interaction on distractor-present trials (shown in Fig. 6D).

To establish whether WM representation fidelity differed across distractor conditions within each ROI and epoch (Fig. 5), we used a slightly different shuffling procedure. Computing WM fidelity on distractor-present trials cannot occur on single trials, and instead we must aggregate across an equivalent number of trials for each relative distractor position. Each scanning run (10 trials) involved one trial for each relative distractor position, so we were able to compute a mean WM representation fidelity for distractor-absent trials (3 trials) and distractor-present trials (7 trials) on each scanning run (26–36 runs per participant). First, we computed a *t*-statistic from a paired *t*-test for each ROI and epoch to quantify the effect of distractor condition on WM fidelity. Then, we computed a null *t*-distribution after shuffling run-wise average WM fidelity scores within each participant 1000 times. P-values were computed as the proportion of the *t*-distribution exceeding the t-statistic computed using intact data and doubled to reflect a two-tailed test. We corrected *p*-values using the false discovery rate across all ROIs and epochs.

To determine whether decoded WM target position just before response onset predicted behavioral response positions on a trial-by-trial basis on distractor-present trials, we extracted decoded WM positions from each ROI (Eq. 6) on each trial with a nearby distractor (within 12° polar angle) and correlated, within each participant, these values with corresponding behavioral responses (Fig. 7a, b). We converted individual Pearson correlation coefficients to Z-scores using the Fisher r-to-Z transform before combining across participants (Fig. 7c) and computed a *t*-score for each ROI. To assess significance, we shuffled the relationship between behavioral and neural responses within each participant 1000 times, recomputed correlations for each participant and *t*-scores across participants, then compared the true *t*-score for each ROI to the corresponding shuffled null distribution. *P*-values (one-tailed) were corrected across ROIs with FDR. We also tested for a main effect of ROI on average trial-by-trial error correlation across participants using a shuffled 1-way ANOVA. To follow-up the individual trial-by-trial analysis, we performed an additional correlation analysis across participants (Fig. 7d; single ROIs Supplementary Fig. 6B). For this analysis, within each participant, neural decoding error was used to sort corresponding behavioral error for that participant into quartiles. One mean was taken per quartile per participant (Fig. 7d). We performed a correlation using the combined data from all participants and compared the correlation coefficient obtained from doing so to a null distribution of correlation coefficients obtained by shuffling the correspondence between neural and behavioral error prior to binning. One-tailed *p*-values for each ROI were defined as the proportion of shuffled coefficients that were greater than the coefficient computed from intact data, corrected for multiple comparisons across ROIs via FDR.

### Reporting summary

Further information on research design is available in the Nature Research Reporting Summary linked to this article.

## Supplementary information

Supplementary Information

Reporting Summary

## Data Availability

The processed fMRI data and raw behavioral and eyetracking data generated in this study have been deposited in the Open Science Framework https://osf.io/c9fst/. Processed fMRI data contains extracted timeseries from each voxel of each ROI. The raw fMRI data are available under restricted access to ensure participant privacy; access can be obtained by contacting the corresponding authors. The data used to plot figures in this paper (participant means) are provided in the Source Data file. Source data are provided with this paper.

## Source data

Source Data

## Code availability

The code used to present stimuli, analyze data and produce figures has been made freely available on GitHub (github.com/clayspacelab). Please cite as: Grace Hallenbeck, Thomas Sprague, Masih Rahmati, Kartik Sreenivasan, and Clayton Curtis. (2021, June 30). clayspacelab/spDist_mFiles: analysis code (Version 1.0.0). Zenodo. 10.5281/zenodo.5048438.
