# Supplementary material for: Working memory representations in visual cortex mediate distraction effects
Source: Nat Commun. 2021 Aug 5;12:4714. doi: 10.1038/s41467-021-24973-1 (PMC8342709; doi:10.1038/s41467-021-24973-1)
Supplement: Supplementary file 1 — Supplementary Information [file 41467_2021_24973_MOESM1_ESM.pdf]

SUPPLEMENTARY MATERIALS FOR:

Working Memory Representations in Visual Cortex Mediate Distraction Effects

Hallenbeck, Sprague, Rahmati, Sreenivasan, & Curtis

SUPPLEMENTARY FIGURES

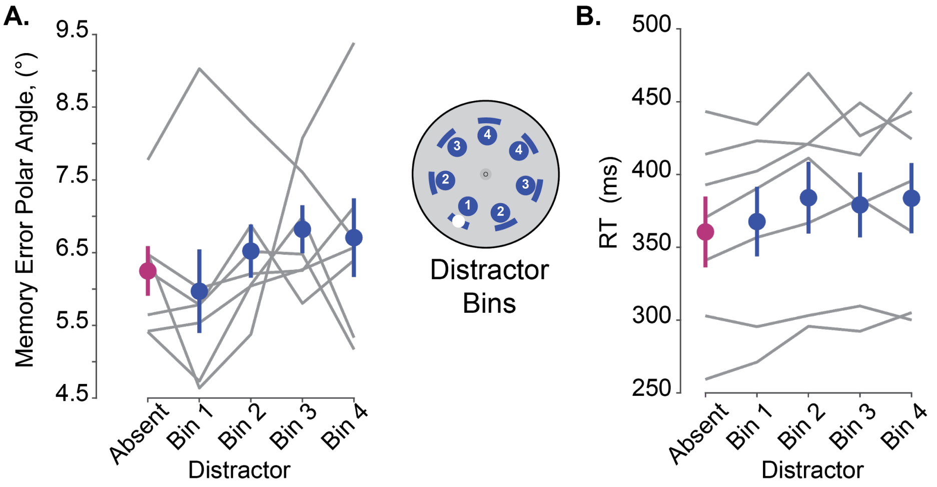

Supplementary Figure 1. **Behavioral performance does not vary across distractor offsets. a** Memory error (group mean, error bars ±SEM) on distractor absent trials (magenta) and memory error per each pseudo-randomized distractor offset bin (blue; each bin 24° wide): bin 1, 0° mean offset; bin 2, 51.42°; bin 3, 102.9°, bin 4, 154.3°, shown at inset. Gray lines depict individual participant (n =7) performance across distractor-absent (30% of trials) and each distractor present condition (each offset comprised 20% of trials, except for bin 1, which was 10%). To determine if the distractor-present offsets differed from one another significantly, we performed a one-way RM ANOVA on only the distractor-present conditions, and found no significant effect of memory error ($F(3,6)=0.84$, $p=0.49$). **b** Saccadic reaction time (group mean, error bars ±SEM) measured from the onset of the initial ballistic saccade from the start of the response cue period, was greater in each distractor condition (blue) as compared to distractor-absent trials. Gray lines depict individual participant (n =7) performance. To determine whether reaction time with respect to each distractor offsets differed significantly from one another, we performed a one-way RM ANOVA. RT did not vary across distractor bins ($F(3,6) = 2.01$, $p=0.15$).

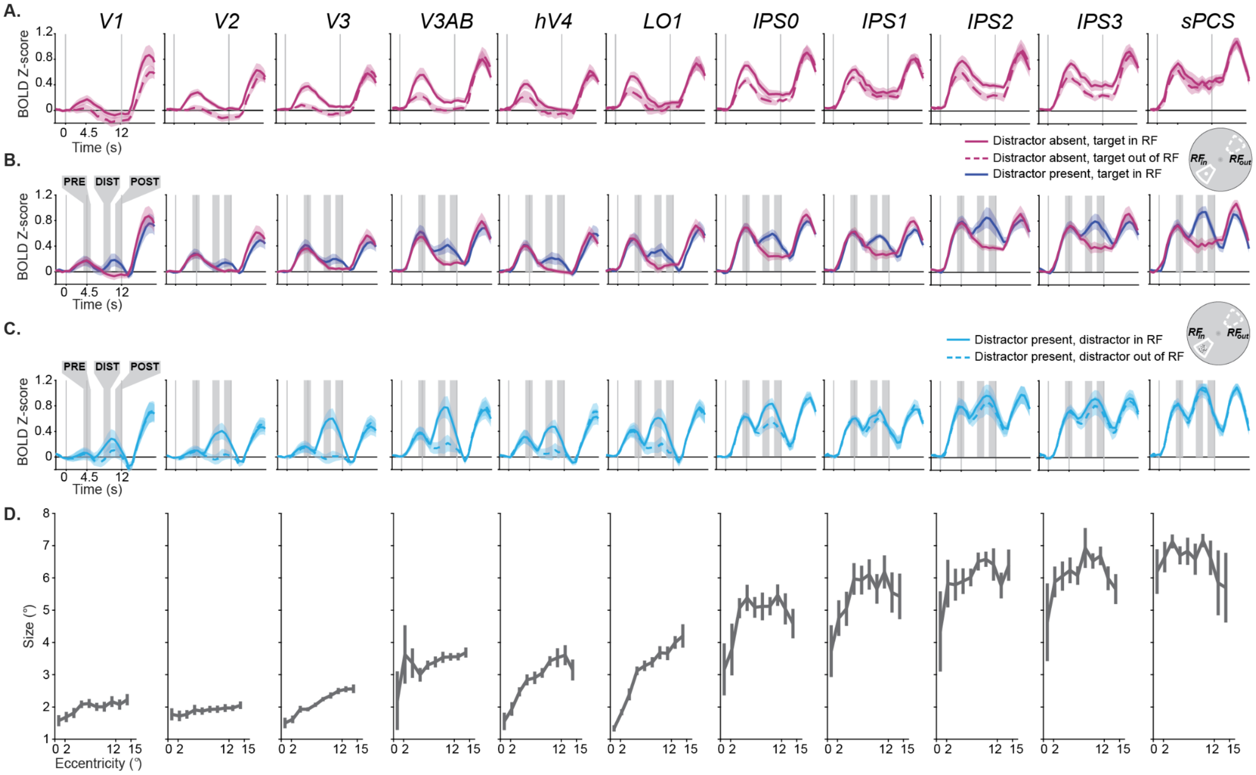

Supplementary Figure 2. BOLD responses sorted by voxel RF position during WM delay period. **a** During distractor-absent trials, the average (±SEM) amplitude of BOLD responses was greater in voxels whose receptive fields aligned with the WM target (RF_{in}) compared to when the target was 180 degrees away from voxels' receptive fields (RF_{out}). The inset to the right depicts an example of the RF_{in} and RF_{out} in respect to the WM target (see Methods for more details). The amplitudes of persistent activity increased moving anterior in the dorsal stream ROIs from early visual cortex to parietal cortex to frontal cortex, while the spatial selectivity decreased. **b** During distractor-present trials, we observed an additional phasic response time-locked to the distractor onset across all ROIs. **c** To further illustrate the distractor response, we averaged the BOLD responses in voxels whose RFs were aligned to the distractor position, regardless of the position of the WM target. The phasic responses were more robust in voxels with RFs that matched (RF_{in}) compared to opposite to the distractor (RF_{out}). The shaded areas denote the pre-distractor, distractor, and post-distractor epochs that are the target of later analyses. **d** Average voxel receptive field properties (±SEM, size plotted binned by eccentricity) in each ROI. Note two trends: the pRF size increases with eccentricity and the size increases as one moves up the hierarchy.

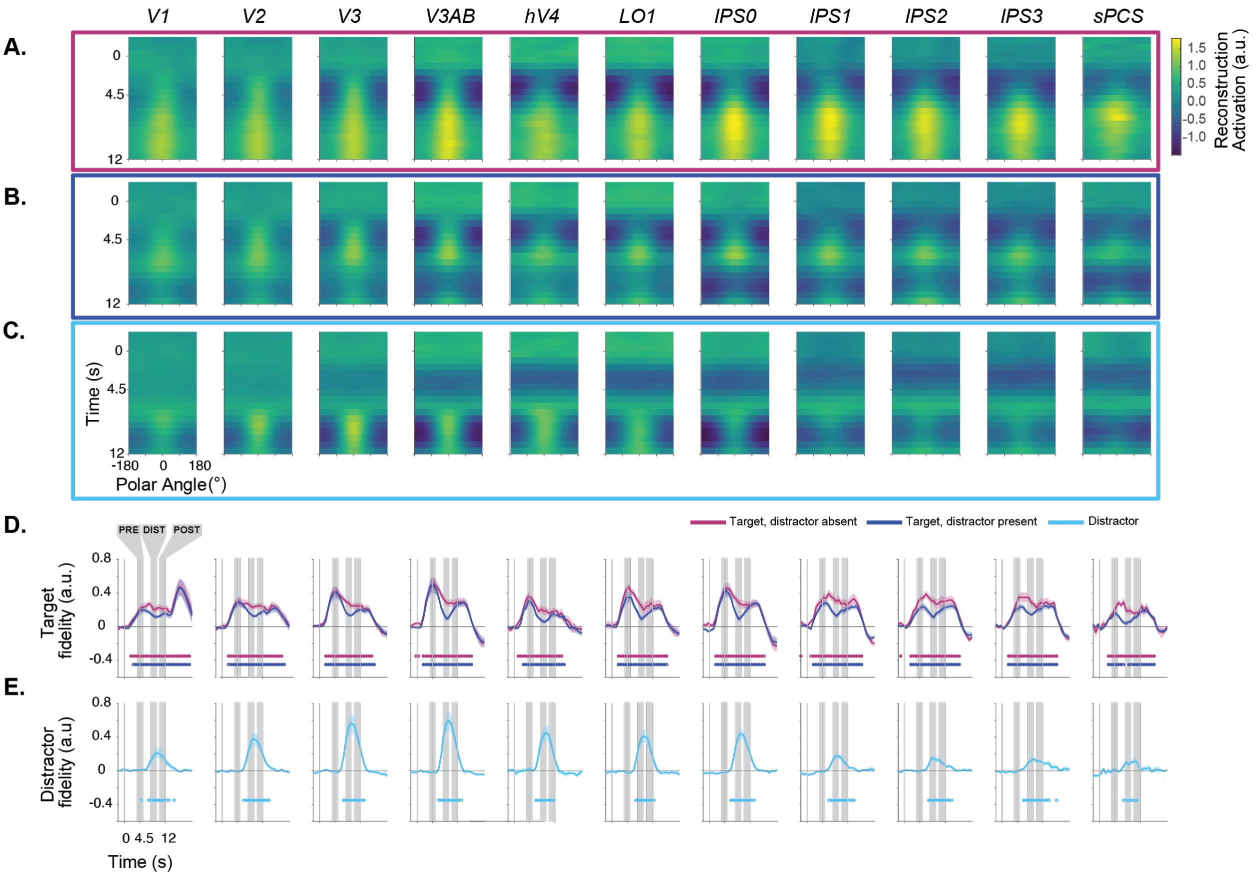

Supplementary Figure 3. Impact of distraction on the dynamics of WM representations in individual ROIs. Data presented as in Figure 3, but for individual ROIs. Average reconstruction of WM target positions on distractor-absent trials (**a**) and distractor-present trials (**b**) across all participants (n=7). **c** Reconstruction of distractor position on distractor-present trials, where all trials were aligned to a fixed distractor position. Note that **b** and **c** are reconstructions of the same data, just aligned to different positions. Reconstruction strength is greatest at the aligned position in each instance and represents the polar angle position of the WM target maintained over the entire delay period or the briefly presented distractor. (**d & e**). Fidelity of the neural representation of WM targets (**d**) and distractors (**e**). When activation peaks in the direction of the remembered target (after alignment), fidelity is positive; when there is no consistent activation peak, fidelity is near zero. Target fidelity on distractor-absent trials is robust and statistically significant throughout the delay period in all ROIs. When the distractor is present, fidelity drops, but remains significantly above zero for all ROIs except sPCS. Distractor fidelity is also statistically significant in all regions and is qualitatively most robust across extrastriate visual cortex (e.g., V3AB). Closed and open circles denote significance of $p<0.05$, one-sided, FDR corrected and $p<0.05$, one-sided, FDR uncorrected, respectively (one-sample t-test using null distribution derived from shuffled IEM; see Methods). Error bars ±SEM.

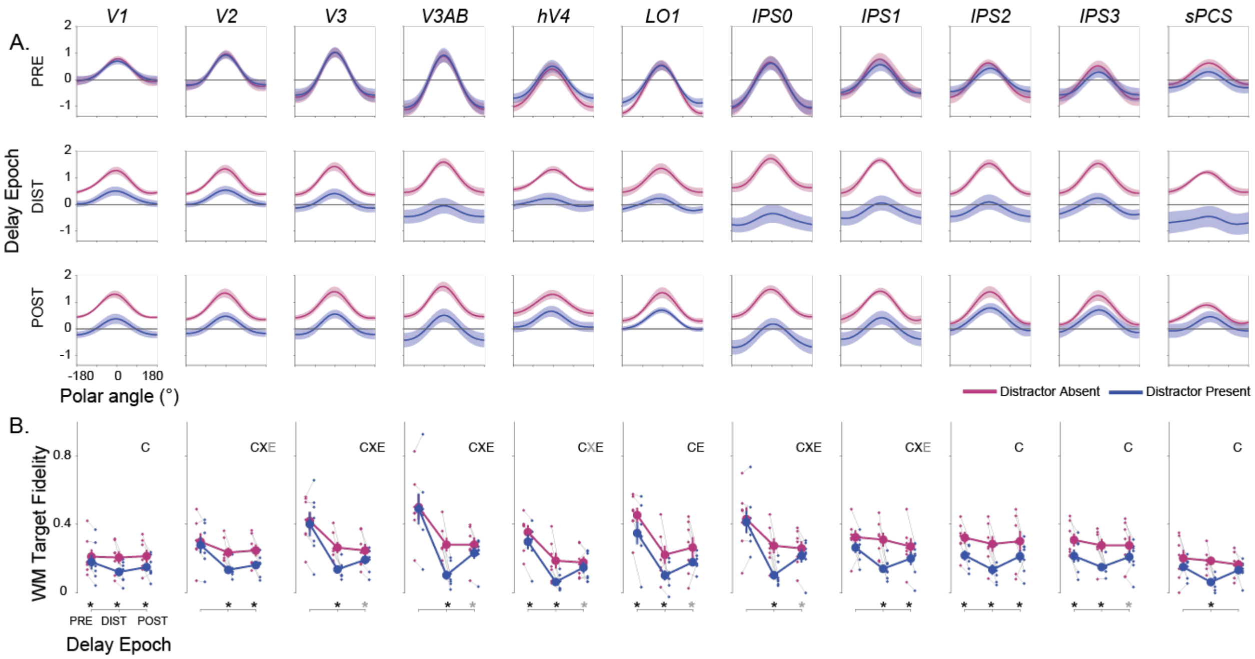

Supplementary Figure 4. WM representations are transiently disrupted by an attended distractor stimulus in individual ROIs. Data presented as in Figure 5, but for individual ROIs. **a** Independently trained model-based reconstructions of the WM target locations on distractor-absent (magenta) and distractor-present trials (blue) estimated separately for three epochs of the memory delay. The epochs were composed of TRs before the distractor (3.75-5.25s), during the distractor (8.25-9.75s), and after the distractor (10.5-12s). Error bars SEM. Note that during the distractor epoch, the reconstructions of the WM target locations appear weaker on distractor-present compared to distractor-absent trials. In some regions, this effect of the distractor lasts into the post-distractor epoch. **b** Average (+/-SEM) fidelity of reconstructed WM targets on distractor-absent (magenta) and distractor-present (blue) trials separately for the pre-distraction, distraction, and post-distraction epochs. Thin gray lines connect mean distractor-absent (small magenta dots) and distractor-present (small blue dots) for individual participants for each delay epoch. The results from 2-way ANOVAs for each ROI (epoch and condition as factors; compared against a shuffled null) are marked by symbols to denote the significant main effects of condition (C), epoch (E), and the interaction between epoch and condition (X). The significant results of paired *t*-tests between distractor-present and distractor-absent reconstructions per epoch, for each ROI, are marked with asterisks. In both cases, gray symbols denote $p<0.05$, uncorrected, and black $p<0.05$, FDR corrected across ROIs. All *p*-values available in Supplementary Table 3.

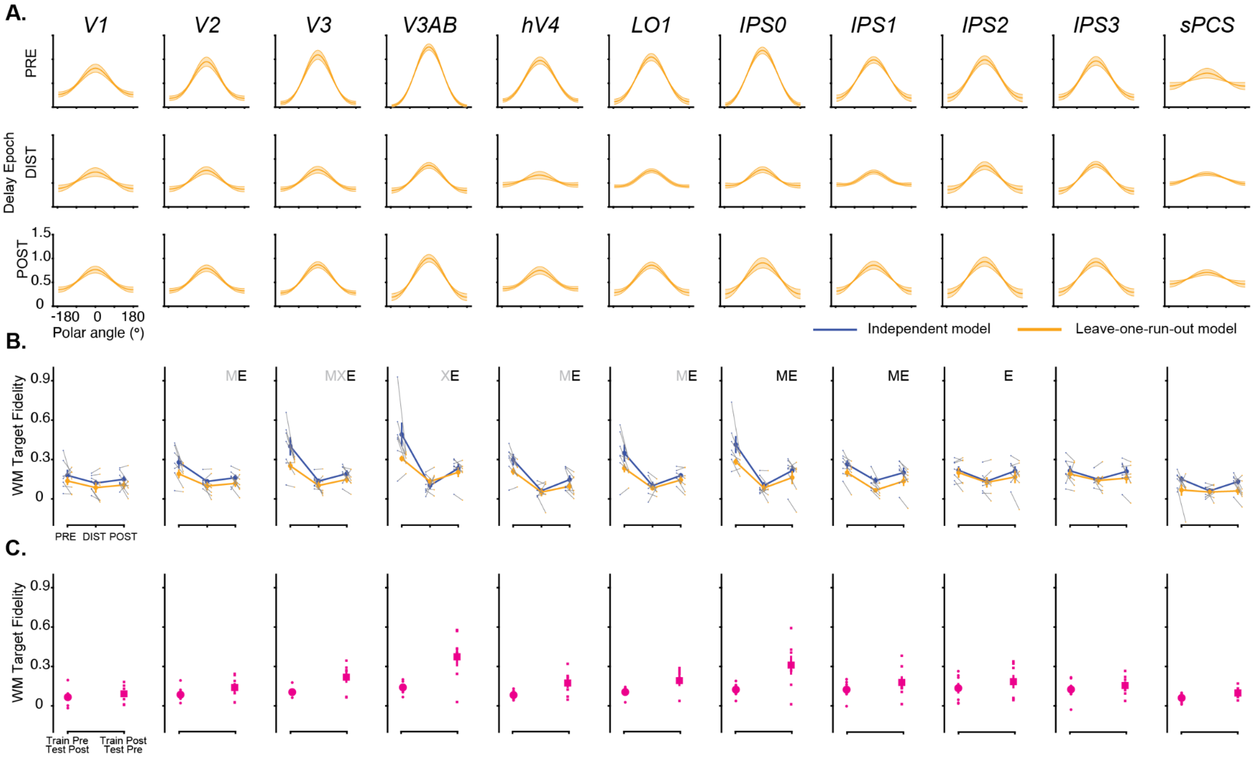

Supplementary Figure 5. Loss of WM fidelity during distraction cannot be explained by a different coding format in individual ROIs. Data presented as in Figure 6, but for individual ROIs. **a** Model-based reconstructions from a cross-temporal generalization analysis in which training and testing was performed on corresponding epochs of the delay (i.e., train IEM with PRE timepoints, reconstruct using PRE timepoints from trials in held-out run). Rows show reconstructions from each ROI from each epoch (error bars ±SEM). Qualitatively, a substantial dip in WM reconstruction strength is apparent during the DIST epoch, as in Supplementary Figure 5a. **b** Comparison of mean (error bars ±SEM) fidelity during each trial epoch across model estimation procedures. Blue line shows data computed using an independent model (replotted from Supplementary Figure 5b); orange line shows data computed using the leave-one-run-out cross-validation procedure. Gray lines show individual participants (n=7). We performed a 2-way repeated measures ANOVA against a shuffled null for each ROI (factors model and trial epoch). Main effects of model are indicated by M, main effects of epoch are indicated by E, and interactions are indicated by X. Significant tests are shown in black ($p < 0.05$, FDR corrected across ROIs within test); trends are shown in gray ($p<0.05$, no correction). Error bars ±SEM. IPS0 & IPS1 show a significant main effect of 'model', though the independently trained model out-performs the leave-one-run-out model. No ROIs show a significant interaction between model and epoch (though a trend is seen in V3 & V3AB, which is largely driven by stronger WM target representations measured using the independent model). *p*-values for all tests available in Supplementary Table 6. **c.** Comparison of off-diagonal training-testing combinations, group means (n=7, error bars ±SEM). To determine if models trained and tested on non-matched epochs were able to recover information, we measured fidelity from models trained on PRE and tested on POST, as well as trained on POST and tested on PRE delay epochs. All *p*'s < 0.05 when comparing each average fidelity against a null distribution of fidelities.

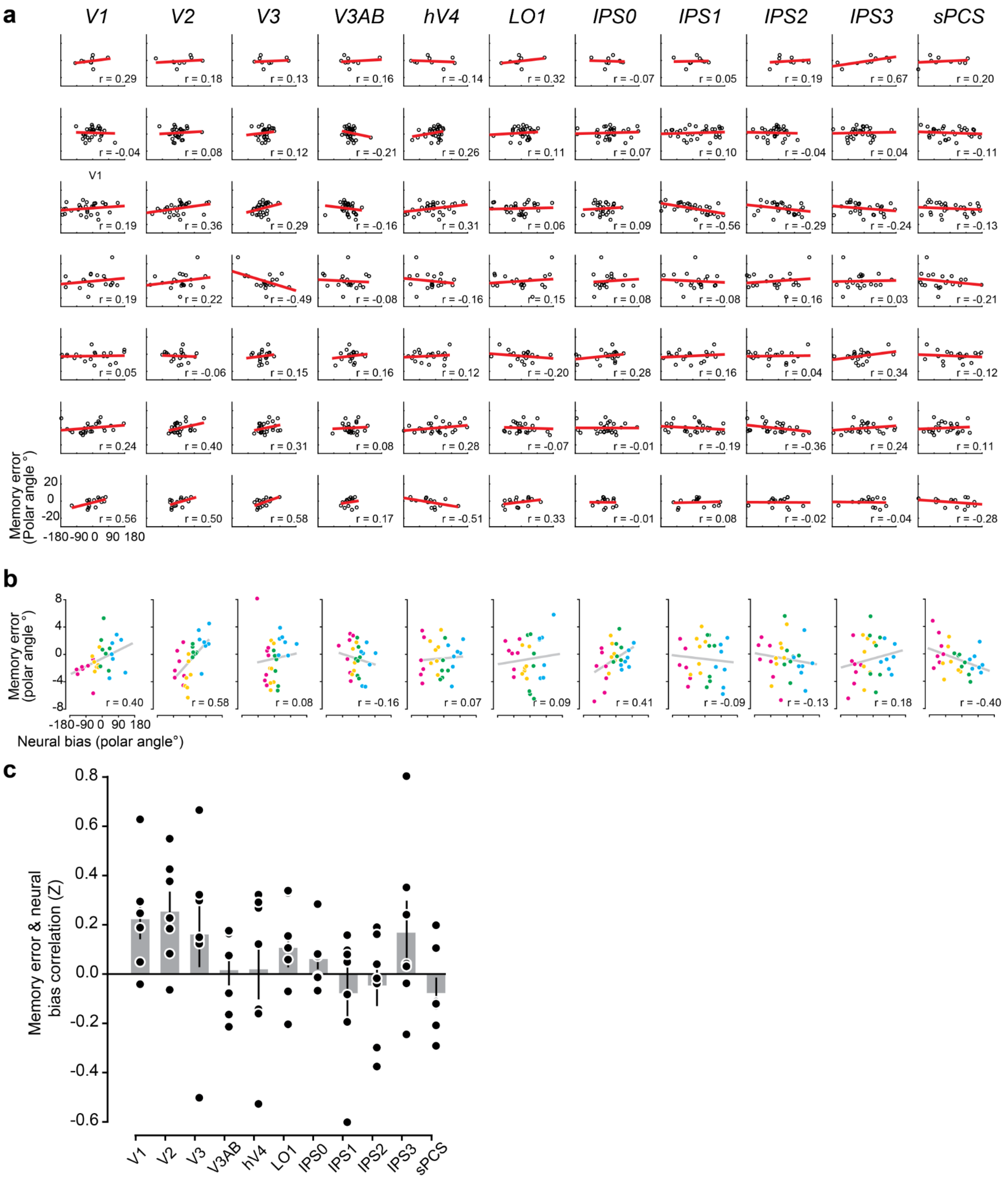

Supplementary Figure 6. Comparison between behavioral errors and neural errors for single ROIs. **a** Correlation between decoded WM representation error and behavioral WM response for each participant and individual ROI separately for distractor-present trials with distractor presented within 12° polar angle of WM target location. **b** Trial-binned correlations as computed in Figure 7. **c** We aggregated the trial-by-trial correlation of each participants' behavioral error with their corresponding neural error across participants (n=7, bar reflects averaged Fisher-Z transformed, error bars ±SEM) and compared these values against 0 (1-way *t*-test against a shuffled null). At the individual ROI level, no significant correlations were found after correction for multiple comparisons (FDR), but trends were observed in V1 and V2 (uncorrected, $p<0.05$). A 1-way repeated-measures ANOVA did not identify a significant main effect of ROI on neural/behavioral error correlations ($p=0.12$; comparison against shuffled null). All *p*-values available in Supplementary Table 7.

SUPPLEMENTARY TABLES

Supplementary Table 1. Non-parametric *p*-values characterizing differences in mean delay period amplitude and RF conditions across grouped ROIs and selected voxel characteristics, Figure 2. a. To test for differences in amplitude across ROIs and reliable differences in RF conditions across dorsal ROIs, a 2-way shuffled ANOVA was performed on mean delay-period amplitude from distractor-absent trial with ROI and RF conditions as main effects. **b**. A follow-up 1-way shuffled ANOVA was performed within each ROI with RF condition (in vs. out) as factors.

a.

**ROI**	**RF**	**ROI x RF**
**<0.001**	**0.001**	**0.035**

b.

**ROI**	**RF**
V1-V3	**0.005**
V3AB	**0.019**
hV4	**0.009**
LO1	**0.012**
IPS0/1	**0.016**
IPS2/3	**0.016**
sPCS	**0.008**
FDR Thresh	0.019

c. Voxel characteristics. Mean number voxels selected per region and their average pRF fit sigma & eccentricity parameters. Averaged over trials within participant and across participants. In 'target-aligned', both conditions (distractor absent & present) are used in averaging. RF-in/RF-out are similar because locations are fully randomized.

**ROI**	**Target-aligned # voxels RF-in, RF-out**	**Distractor-aligned # voxels RF-in, RF-out**	**Target-aligned avg. pRF sigma & ecc RF-in; RF-out**	**Distractor-aligned avg. sigma & ecc RF-in; RF-out**
**V123**	**187.01, 187.78**	**187.92, 186.00**	**2.00, 7.26; 2.01, 7.26**	**1.98, 7.20 ; 1.99, 7.23**
**V3AB**	**38.83, 37.90**	**35.72, 36.44**	**3.51,9.16; 3.51,9.21**	**3.51, 9.17; 3.52, 9.18**
**hV4**	**22.05, 22.34**	**22.63, 22.04**	**2.59, 5.21; 2.61,5.26**	**2.57, 5.11; 2.56, 5.18**

LO1	22.71, 22.28	21.81, 21.97	3.06,5.91;<br>3.06,5.75	3.11, 5.86 ;<br>3.10, 5.85
IPS0/1	28.97, 28.21	26.97, 28.11	5.01,8.67;<br>5.05, 8.68	5.01, 8.73;<br>5.03, 8.64
IPS2/3	25.17, 25.46	24.16, 25.01	6.02,8.44;<br>6.00, 8.43	5.94, 8.46 ;<br>5.97,8.45
sPCS	15.36, 15.39	14.61,14.90	6.34,6.88;<br>6.35, 7.06	6.32, 6.81;<br>6.33, 6.89

Supplementary Table 2. Non-parametric *p*-values characterizing differences in mean delay period amplitude and RF conditions (a-b), as well as differences between conditions across delay epochs (c-f) in individual ROIs, Supplementary Figure 2. a. To test for differences in amplitude across ROIs and reliable differences in RF conditions across dorsal ROIs, a 2-way shuffled ANOVA was performed on mean delay-period amplitude from distractor-absent trial with ROI and RF conditions as main effects. **b**. A follow-up 1-way shuffled ANOVA was performed within each ROI with RF condition (in vs. out) as factors. Significant tests are marked in bold; trends (p<0.05, uncorrected) are marked in italics. **c.** 3-way ANOVA was performed on RF-in data with ROI, condition, & epoch as main effects. **d.** 2-way permuted ANOVA tests on RF-in data per ROI with condition (distractor absent vs. present) and epoch as main effects. **e.** 3-way ANOVA was performed in RF-in-out data with ROI, condition, & epoch as main effects. **f.** 2-way permuted ANOVA tests on RF-in-out data per ROI with condition (distractor absent vs. present) and epoch as main effects. Significant tests are marked in bold; trends (p<0.05, uncorrected) are marked in italics.

a.

ROI	RF	ROI x RF
**<0.001**	**0.001**	**0.035**

b.

ROI	RF
V1	**0.030**
V2	**0.006**
V3	**0.006**
V3AB	**0.017**
hV4	**0.003**
LO1	**0.007**
IPS0	**0.028**
IPS1	0.064
IPS2	**0.008**
IPS3	**0.015**
sPCS	**0.011**
FDR Threshold	0.030

c.

ROI	COND	EPOCH	ROI X COND	ROI X EPOCH	COND X EPOCH	ROI X COND X EPOCH
**0**	0.042	**0**	0	**0**	0	**0**

d.

ROI	COND	EPOCH	COND X EPOCH
V1	**0.007**	**0**	**0**
V2	0.262	**0**	**0.039**
V3	0.135	**0**	0.1
V3AB	*0.046*	**0**	**0.008**
hV4	0.065	**0**	**0.003**
LO1	0.121	**0**	**0.001**
IPS0	*0.022*	**0**	**0**
IPS1	*0.037*	**0**	**0.002**
IPS2	**0.012**	**0**	**0**
IPS3	0.077	**0.019**	**0**
sPCS	**0.006**	**0.014**	**0**
FDR THRESH	0.012	0.019	0.039

e.

ROI	COND	EPOCH	ROI X COND	ROI X EPOCH	COND X EPOCH	ROI X COND X EPOCH
**0**	0.041	**0**	0.927	**0**	0.061	0.067

f.

ROI	COND	EPOCH	COND X EPOCH
V1	0.097	0.06	0.09
V2	**0.002**	**.001**	**0.009**
V3	*0.027*	**0**	**0**

V3AB	0.285	**0**	**0**
hV4	0.357	**0**	**0.004**
LO1	0.856	**0**	0.654
IPS0	0.143	**0**	0.051
IPS1	0.432	**0.01**	0.981
IPS2	0.244	**0.015**	0.328
IPS3	0.148	0.312	0.654
sPCS	0.645	0.065	0.479
FDR THRESH	0.002	0.015	0.009

Supplementary Table 3. Non-parametric *p*-values characterizing effect of distraction on WM representation fidelity (individual ROIs), Supplementary Figure 4.
p-values for 3-way, 2-way, and *t*-tests per each ROI from Figure S4. For each test, test statistics (F- or T-scores) computed using intact data labels were compared against those computed using data labels shuffled within each participant 1000x.

a. P-values from permuted 3-way ANOVA. Bold indicates significant tests.

**Epoch**	**Cond**	**ROI**	**Epoch × Cond**	**Epoch × ROI**	**Cond × ROI**	**Epoch × Cond × ROI**
**< 0.001**	**0.002**	**< 0.001**	**0.001**	**< 0.001**	0.479	**0.028**

b. P-values from permuted 2-way ANOVA with distractor condition (absent / present) and delay epoch (pre,dist,post) as main effects for each ROI. FDR corrections were applied across ROIs for each effect. Bold indicates significant tests (FDR-corrected); italics indicates trends, defined as $p < 0.05$, uncorrected.

	**Epoch**	**Condition**	**Epoch x Condition**
V1	0.689	**0.004**	0.202
V2	*0.05*	**0.007**	**0.006**
V3	**0.001**	**0.005**	**< 0.001**
V3AB	**< 0.001**	**0.005**	**< 0.001**
hV4	**0.001**	**0.008**	*0.048*
LO1	**0.003**	**0.017**	0.722
IPS0	**< 0.001**	**0.005**	**0.004**
IPS1	**0.023**	**0.017**	*0.031*
IPS2	0.109	**0.001**	0.358
IPS3	0.388	**0.004**	0.272

sPCS	0.361	**0.033**	0.089
FDR Threshold	0.023	**0.033**	0.006

c. To directly compare WM representation fidelity between distractor present vs distractor absent trials, we computed fidelity for each condition averaged across all trials within each run, then averaged across runs within participant and performed a *t*-test on the resulting set of data points per participant. To generate a null *t*-distribution, we randomly shuffled condition labels per run before averaging 1000x. The T-score estimated using intact data was compared against these shuffled null distributions (two-tailed) to derive a *p*-value (listed below). Bold values indicate significant tests (FDR-corrected within each epoch), italics indicates trends defined as $p < 0.05$, uncorrected.

	**Epoch 1 (PRE)**	**Epoch 2 (DIST)**	**Epoch 3 (POST)**
V1	**0.0260**	**0.0120**	**0.0060**
V2	0.1160	**0.0020**	**0.0080**
V3	0.1600	**< 0.001**	*0.0360*
V3AB	0.2340	**0.0020**	*0.0360*
hV4	**0.0160**	**0.0040**	*0.0500*
LO1	**0.0040**	**0.0180**	*0.0420*
IPS0	0.1940	**< 0.001**	*0.0460*
IPS1	0.0660	**0.0020**	**0.0140**
IPS2	**0.0020**	**< 0.001**	**0.0300**
IPS3	**0.0020**	**< 0.001**	*0.0500*
sPCS	0.0820	**0.0020**	0.1420
FDR THRESH	0.03	**0.03**	0.03

Supplementary Table 4. Non-parametric *p*-values characterizing effect of distraction on WM representation fidelity, Figure 5b. *p*-values for 3-way, 2-way, and *t*-tests per each ROI from Figure 5B. For each test, test statistics (F- or T-scores) computed using intact data labels were compared against those computed using data labels shuffled within each participant 1000x.

a. P-values from permuted 3-way ANOVA. Bold indicates significant tests.

**Epoch**	**Condition**	**ROI**	**Epoch x Condition**	**Epoch x ROI**	**Cond x ROI**	**Epoch x Condition x ROI**
**< 0.001**	**0.001**	**< 0.001**	**0.001**	**< 0.001**	0.551	**0.041**

b. P-values from permuted 2-way ANOVA with distractor condition (absent / present) and delay epoch (pre,dist,post) as main effects for each ROI. FDR corrections were applied across ROIs for each effect. Bold indicates significant tests (FDR-corrected); italics indicates trends, defined as *p*<0.05, uncorrected.

	**Epoch**	**Condition**	**Epoch x Condition**
V1-V3	**0.013**	**0.011**	**0.002**
V3AB	**0**	**0.004**	**0.001**
hV4	**0.001**	**0.008**	*0.044*
LO1	**0.002**	**0.011**	0.747
IPS0/1	**0.001**	**0.002**	**0.004**
IPS2/3	0.187	**0.002**	0.181
sPCS	0.334	**0.036**	0.087
FDR THRESH	0.013	0.036	0.004

c. To directly compare WM representation fidelity between distractor present vs. distractor absent trials, we computed fidelity for each condition averaged across all trials within each run, then averaged across runs within participant and performed a *t*-test on the resulting set of data points per participant. To generate a null *t*-distribution, we randomly shuffled condition labels per run before averaging 1000x. The T-score estimated using intact data was compared against these shuffled null distributions (two-tailed) to derive a *p*-value (listed below). Bold values indicate

significant tests (FDR-corrected within each epoch), italics indicates trends defined as $p < 0.05$, uncorrected.

	**Epoch 1 (PRE)**	**Epoch 2 (DIST)**	**Epoch 3 (POST)**
V1-V3	0.076	**0.004**	**0.012**
V3AB	0.218	**0.002**	**0.022**
hV4	**0.026**	**0.004**	0.056
LO1	**0.006**	**0.01**	*0.042*
IPS0/1	0.096	**0.002**	**0.006**
IPS2/3	**< 0.001**	**< 0.001**	**0.03**
sPCS	0.124	**< 0.001**	0.112
FDR THRESH	0.03	0.03	0.03

Supplementary Table 5 (comparison of WM fidelity on distractor-present trials across model estimation procedures). **a.** A 3-way ANOVA was performed with ROI, model, & epoch as factors **b.** For each ROI, we performed a 2-way repeated measures ANOVA against a shuffled null (within each participant, shuffle datapoint labels 1000x). For each test (main effect of model, main effect of epoch, interaction), we compute an FDR threshold. Significant tests are marked in bold; trends (*p*<0.05, uncorrected) are marked in italics.

a. All ROIs 3-way ANOVA:

**ROI**	**Model**	**Epoch**	**ROI × Model**	**ROI × Epoch**	**Model × Epoch**	**ROI × Model × Epoch**
**< 0.001**	**0.005**	**<0.001**	0.326	**<0.001**	0.055	**0.001**

b. 2-way repeated measures ANOVA:

**Supplementary Figure 5c**	**Model**	**Epoch**	**Interaction**
V1	0.147	0.259	0.934
V2	0.031	**0.003**	0.238
V3	*0.023*	**<0.001**	*0.015*
V3AB	0.054	**<0.001**	*0.021*
hV4	*0.02*	**<0.001**	0.179
LO1	*0.018*	**<0.001**	0.075
IPS0	**0.009**	**0.001**	0.121
IPS1	**<0.001**	**0.010**	0.866
IPS2	0.239	**0.036**	0.288
IPS3	0.226	0.146	0.335
sPCS	0.065	0.084	0.176
FDR THRESHOLD	0.009	0.036	<0.001

Supplementary Table 6. Non-parametric *p*-values quantifying impact of model estimation procedures, Figure 6. Statistics for Figure 6D (comparison of WM fidelity on distractor-present trials across model estimation procedures). **a.** We performed a 3-way ANOVA against a shuffled null with main effects of ROI, model, & epoch. Significant tests are marked in bold. **b.** For each ROI, we performed a 2-way repeated measures ANOVA against a shuffled null (within each participant, shuffle datapoint labels 1000x). For each test (main effect of model, main effect of epoch, interaction), we compute an FDR threshold. Significant tests are marked in bold; trends (p< 0.05, uncorrected) are marked in italics.

a.

ROI	Model	Epoch	ROI × Model	ROI × Epoch	Model × Epoch	ROI × Model × Epoch
**< 0.001**	**0.010**	**<0.001**	0.641	**<0.001**	**0.029**	**0.018**

b.

Figure 6d	Model	Epoch	Model x Epoch
V1-V3	*0.037*	**0.001**	0.127
V3AB	*0.047*	**<0.001**	*0.017*
hV4	*0.023*	**<0.001**	0.145
LO1	*0.015*	**<0.001**	0.079
IPS0/IPS1	**0.004**	**<0.001**	0.097
IPS2/IPS3	0.248	*0.047*	0.196
sPCS	*0.048*	0.086	0.212
FDR thresh	0.004	0.001	<0.001

Supplementary Table 7. P-values characterizing neural-behavioral correlations (individual ROIs), Supplementary Figure 6b.

Shuffled correlations were performed on an individual basis and subjected to a *t*-test on Fisher r-to-Z transformed correlation values, 1000x. No ROIs pass FDR correction. Italics indicates trends, defined as p<0.05, uncorrected.

**ROI**	**V1**	**V2**	**V3**	**V3AB**	**hV4**	**LO1**	**IPS0**	**IPS1**	**IPS2**	**IPS3**	**sPCS**
p-value	*0.017*	*0.008*	0.161	.389	.465	.105	.095	.717	.732	.119	.853

Supplementary Table 8. A. P-values characterizing neural-behavioral correlations, Figure 7. Shuffled correlations were performed on an individual basis and subjected to *t*-test with Fisher r-to-Z transformed correlation values, 1000x. FDR threshold is *p*=0.005. Bold indicates significant ROIs, corrected for multiple comparisons.

**ROI**	**V1-V3**	**V3AB**	**hV4**	**LO1**	**IPS0/1**	**IPS2/3**	**sPCS**
Fisher-Z	**0.31**	0.01	0.02	0.10	0.01	0.05	-0.07
p-value	**0.005**	0.4104	0.4710	0.1040	0.4310	0.3130	0.8670

B. Individual participant data from trial-wise correlations.

**Participant**	**rho**	**p-value**	**# trials submitted to correlation out of possible**
1	0.21	0.58	9/26
2	0.01	0.94	31/32
3	0.41	0.02	30/32
4	0.36	0.11	20/33
5	0.04	0.84	21/32
6	0.41	0.03	26/36
7	0.58	0.02	15/35

C. Trial-binned correlation coefficients and corresponding p-values obtained from comparing against a shuffled null, one-tailed, FDR corrected, from Figure 7d.

**ROI**	**V1-V3**	**V3AB**	**hV4**	**LO1**	**IPS0/1**	**IPS2/3**	**sPCS**
r	**0.54**	-0.15	0.06	0.09	0.21	-0.01	-0.40
p-value	**0.007**	0.751	0.508	0.412	0.218	0.398	0.957
