# Supplementary material for: Working memory representations in visual cortex mediate distraction effects
Source: Nat Commun. 2021 Aug 5;12:4714. doi: 10.1038/s41467-021-24973-1 (PMC8342709; doi:10.1038/s41467-021-24973-1)
Supplement: Supplementary file 2 — Reporting Summary [file 41467_2021_24973_MOESM2_ESM.pdf]

nature research

Corresponding author(s): Curtis, CE

Last updated by author(s): Jul 1, 2021

Reporting Summary

Nature Research wishes to improve the reproducibility of the work that we publish. This form provides structure for consistency and transparency in reporting. For further information on Nature Research policies, see our Editorial Policies and the Editorial Policy Checklist.

Statistics

For all statistical analyses, confirm that the following items are present in the figure legend, table legend, main text, or Methods section.

n/a	Confirmed	
☐	☒	The exact sample size (*n*) for each experimental group/condition, given as a discrete number and unit of measurement
☐	☒	A statement on whether measurements were taken from distinct samples or whether the same sample was measured repeatedly
☐	☒	The statistical test(s) used AND whether they are one- or two-sided<br>*Only common tests should be described solely by name; describe more complex techniques in the Methods section.*
☒	☐	A description of all covariates tested
☐	☒	A description of any assumptions or corrections, such as tests of normality and adjustment for multiple comparisons
☐	☒	A full description of the statistical parameters including central tendency (e.g. means) or other basic estimates (e.g. regression coefficient) AND variation (e.g. standard deviation) or associated estimates of uncertainty (e.g. confidence intervals)
☐	☒	For null hypothesis testing, the test statistic (e.g. *F*, *t*, *r*) with confidence intervals, effect sizes, degrees of freedom and *P* value noted<br>*Give P values as exact values whenever suitable.*
☒	☐	For Bayesian analysis, information on the choice of priors and Markov chain Monte Carlo settings
☒	☐	For hierarchical and complex designs, identification of the appropriate level for tests and full reporting of outcomes
☒	☐	Estimates of effect sizes (e.g. Cohen's *d*, Pearson's *r*), indicating how they were calculated

Our web collection on statistics for biologists contains articles on many of the points above.

Software and code

Policy information about availability of computer code

Data collection	Data were collected with code created using Matlab 2018b
Data analysis	fMRI data were preprocessed using AFNI (version 17.3.09) & FreeSurfer (version 6.0). These data were analyzed using custom Matlab code implementing previously-reported analysis techniques. All code used for preprocessing neural data is available at: github.com/tommysprague/preproc_shFiles, github.com/tommysprague/preproc_mFiles, github.com/tommysprague/vistasoft_ts (RF-fitting), github.com/tommysprague/gridfitgpu (GPU acceleration). All analysis code is available at: github.com/clayspacelab/spDist_mFiles. All code used to preprocessed and analyze eye-tracking data is available at: github.com/clayspacelab/iEye/tree/iEye_ts.

For manuscripts utilizing custom algorithms or software that are central to the research but not yet described in published literature, software must be made available to editors and reviewers. We strongly encourage code deposition in a community repository (e.g. GitHub). See the Nature Research guidelines for submitting code & software for further information.

Data

Policy information about availability of data

All manuscripts must include a data availability statement. This statement should provide the following information, where applicable:

- Accession codes, unique identifiers, or web links for publicly available datasets
- A list of figures that have associated raw data
- A description of any restrictions on data availability

Data Availability
The processed fMRI data and raw behavioral and eyetracking data generated in this study have been deposited in the Open Science Framework https://osf.io/c9fst/. Processed fMRI data contains extracted timeseries from each voxel of each ROI. The raw fMRI data are available under restricted access to ensure participant privacy; access can be obtained by contacting the corresponding authors. The data used to plot figures in this paper (participant means) are provided in the Source

Data file.

Field-specific reporting

Please select the one below that is the best fit for your research. If you are not sure, read the appropriate sections before making your selection.

☐ Life sciences ☒ Behavioural & social sciences ☐ Ecological, evolutionary & environmental sciences

For a reference copy of the document with all sections, see nature.com/documents/nr-reporting-summary-flat.pdf

Behavioural & social sciences study design

All studies must disclose on these points even when the disclosure is negative.

Study description	This study measured human participants' working memory performance during a difficult task with an attended distractor with fMRI & high-speed eye-tracking. The data were measured quantitatively.
Research sample	Seven human participants (3 female) between 25-50 years old participated in the experiment. The same participants participated in independent functional mapping tasks in which retinotopic data was collected, as well as independent single-item memory guided saccade scans which were used to train multivariate models, on separate occasions. The participants were chosen randomly, and are representative of the general population (2 White Male, 1 White female, 1 Latina female, 1 Asian female, 1 Asian male, 1 Persian Male).
Sampling strategy	The sample was determined by comparing our study with previously published work that relied on similar multi-session fMRI scanning techniques and independent mapping data. (Rademaker et al., 2019, n=6; Sprague et al., 2016, n=6)
Data collection	Stimuli were presented and behavioral responses were collected on a Dell PC running Windows 7 & Matlab 2018b. fMRI data were collected Siemens Prisma 3T scanner. Eyetracking data were measured using an in-scanner SR Research Eyelink infrared video-based eyetracker (500 Hz). For each run of the experiment, trials (distractor or no distractor) were presented in randomized order. Participants and experimenters were not blinded to the condition on each trial. Only the experimenter and the participant were present during data collection.
Timing	Data for the main experimental task were first collected on 8/30/2018 and last collected on 03/28/2019. Anatomical images, retinotopic mapping, and independent spatial mapping data were acquired earlier for some participants.
Data exclusions	No data were excluded outright from fMRI or eye-tracking analysis. Upon analysis of eye-tracking data, any data that met specified exclusion criteria were excluded for behavioral analyses. The exclusion criteria used to eliminate behavioral data and consequently neural data were the following: 1. Saccade exceeded length total duration of 150ms and was not at least 5° in amplitude 2. Saccade was not within at least 5° error from the target location. 3. Participant exhibited a fixation break of at least 2.5° during the WM delay 4. Participant did not make a saccade within the specific response epoch<br>Exclusion criteria were determined blind to the condition of each trial and were applied in a fully automated fashion.
Non-participation	No participants dropped out from the experiment.
Randomization	We analyzed all data on a within-participant basis, therefore we did not randomize any aspect of data collection with respect to any given participant.

Reporting for specific materials, systems and methods

We require information from authors about some types of materials, experimental systems and methods used in many studies. Here, indicate whether each material, system or method listed is relevant to your study. If you are not sure if a list item applies to your research, read the appropriate section before selecting a response.

Materials & experimental systems

n/a	Involved in the study	
☒	☐	Antibodies
☒	☐	Eukaryotic cell lines
☒	☐	Palaeontology and archaeology
☒	☐	Animals and other organisms
☐	☒	Human research participants
☒	☐	Clinical data
☒	☐	Dual use research of concern

Methods

n/a	Involved in the study	
☒	☐	ChIP-seq
☒	☐	Flow cytometry
☐	☒	MRI-based neuroimaging

Human research participants

Policy information about studies involving human research participants

Population characteristics	Seven neurologically healthy volunteers (3 female; aged 25-50 years old) with normal or corrected-to-normal vision participated in our study. This sample comprised of members of the NYU graduate student, post-doc, and faculty communities. One participant was an author.
Recruitment	Participants were recruited through word of mouth within the department, and non-lab members received remuneration at $30/hour for their participation. All participants had previously enrolled in fMRI studies within the lab. Within this sample, it is important to note that most of the participants are highly-trained, and this may impact the quality of our results. The way we anticipate this affecting our result is that these individuals may make fewer errors overall.
Ethics oversight	This study was performed at the Center for Brain Imaging at New York University and approved by the NYU institutional review board (IRB).

Note that full information on the approval of the study protocol must also be provided in the manuscript.

Magnetic resonance imaging

Experimental design

Design type	In the experimental and independent mapping scans, a slow event-related design was used. In the functional localizer (retinotopic mapping) scans, a population receptive field approach was used whereby we averaged fMRI timeseries within each voxel across several repetitions of a visual stimulus sequence, then fit a predictive parametric model to each voxel's timeseries (see Mackey et al, 2017).
Design specifications	Within one block, participants performed 3 distractor absent and 7 distractor present trials. In total, the trial length was 24, 27, or 30 seconds. Between trials, the intertrial interval (ITI) was 7,10, or 13 seconds. Participants performed between 12-18 blocks per session, and all participants performed two experimental sessions.
Behavioral performance measures	Correct distractor discrimination per trial and over all trials was recorded and used to compute participant accuracy on the distractor task, which allowed us to change the difficulty of the task across runs by varying dot coherence. For eye-data, saccades were recorded, and from these we computed the distance of the final saccadic endpoint from the target on each run. Across all runs of a given trial type, we took the standard deviation of the polar angle of these final saccades and reported this as memory error. Additionally, we recorded the response time from the response cue to the first eye movement as the saccadic reaction time.

Acquisition

Imaging type(s)	functional
Field strength	3T
Sequence & imaging parameters	BOLD contrast images were acquired using a Multiband (MB) 2D GE-EPI with MB factor of 4, 44 2.5mm interleaved slices with no gap, isotropic voxel size 2.5mm and TE/TR: 30/750ms. We measured field inhomogeneities by acquiring spin echo images with normal and reversed phase encoding (3 volumes each), using a 2D SE-EPI with readout matching that of the GE-EPI and same number of slices, no slice acceleration, TE/TR: 45.6/3537ms. For retinotopic scans, BOLD contrast images were acquired using a Multiband (MB) 2D GE-EPI with MB factor of 4, 56 2mm interleaved slices with no gap, isotropic voxel size 2mm and TE/TR: 42/1300ms. Distortion mapping scans were acquired with normal and reversed phase encoding, using a 2D SE-EPI with readout matching that of the GE-EPI and same number of slices, no slice acceleration, TE/TR: 71.8/6690ms. For anatomical scans, T1- and T2-weighted images were acquired using the Siemens product MPRAGE and Turbo Spin-Echo sequences (both 3D) with 0.8 mm isotropic voxels, 256 × 240 mm slice FOV, and TE/TR of 2.24/2400 ms (T1w) and 564/3200 ms (T2w). We collected 192 and 224 slices for the T1w and T2w images, respectively. We acquired between two and five T1 images, which were aligned and averaged to improve signal-to-noise ratio. In addition, to correct functional images for inhomogeneities in the receive coil sensitivity and improve the motion correction and coregistration process, we collected two fast 3D GRE sagittal images (resolution: 2mm isotropic, FoV: 256 × 256 × 176 mm; TE/TR: 1.03/250 ms), one with the body coil and the other with the 64 ch head/neck coil.
Area of acquisition	Whole brain scans were collected. For some participants, the acquisition sequence did not fully span the ventral aspects of frontal cortex or anterior temporal poles.

Diffusion MRI ☐ Used ☒ Not used

Preprocessing

Preprocessing software	Preprocessing of anatomical images was performed using Freesurfer's recon-all (version 6.0). These were inspected visually and corrected by hand using Freeview, and converted to SUMA format. These anatomical images served as the alignment targets for each participants'' functional data. Preprocessing of functional data was performed using a combination of scripts

generated with AFNI's afni_proc.py and custom scripts implementing AFNI functions (version 17.3.09, pre-compiled Ubuntu 16 64 bit distribution). We performed all analyses on a LINUX workstation running Ubuntu v16.04.1 using 8 cores for most OpenMP accelerated functions. First, we corrected functional images for intensity inhomogeneity induced by the high-density receive coil by dividing all images by a smoothed bias field (15mm FWHM), computed as the ratio of signal in the receive field image acquired using the head coil to that acquired using the in-bore 'body' coil. To improve coregistration of functional data to the target T1 anatomical image, we used distortion-corrected and averaged spin-echo images (which were used to compute distortion fields restricted to the phase-encode direction) to compute transformation matrices between functional and anatomical images. Then, we used the rigorous distortion-correction procedure implemented in afni_proc.py to undistort and motion-correct functional images. Briefly, this procedure involved first distortion-correcting all images in each run using the distortion field computed from the spin-echo image pair, then computing motion-correction parameters (6-parameter affine transform) using these unwarped images. Next, we used the distortion field, motion correction transform for each volume, and the functional-to-anatomical coregistration simultaneously to render functional data from native acquisition space into unwarped, motion corrected, and coregistered anatomical space for each participant at the same voxel size as data acquisition in a single transformation and resampling step. For retinotopic mapping data, this was a 2mm isovoxel grid; and for task data, this was 2.5mm isovoxel grid. For both task and retinotopy data, we projected this volume-space data onto the reconstructed cortical surface. For retinotopy data, we made a smoothed version of the data by smoothing on the cortical surface (5mm FWHM). We then projected surface data (for task data, only the 'raw' data; for retinotopy data, the raw and smoothed data) back into volume space for all analyses. For unsmoothed data, this results in a small amount of smoothing for each voxel along a vector orthogonal to the surface in volume space.

Normalization	Functional data were kept in each participants' native space.
Normalization template	Functional data were kept in each participant's native space.
Noise and artifact removal	see details within 'Preprocessing Software' above.
Volume censoring	No volume censoring was performed.

Statistical modeling & inference

Model type and settings

For our univariate analysis, we computed an event-related average of measured BOLD response for each condition separately. After extracting Z-scored BOLD signalfrom each voxel, we sorted voxels on each trial according to their best-fit pRF parameters and the known location(s) of the target and/or distractor. RF-in responses (corresponding to voxels tuned nearby the relevant location) were determined by selecting voxels with ≥ 10% variance explained, eccentricity between 2 and 15 degrees, and polar angle difference between the WM target (or distractor) and pRF center of each voxel ≤ 15 degrees. RF-out responses were determined by selecting voxels with ≥ 10% variance explained, eccentricity between 2 and 15 degrees, and a polar angle difference between the WM target (or distractor) and pRF center of each voxel ≥ 165 degrees. We averaged responses across such selected voxels within each ROI, then across all trials within a condition (Figure 2, all ROIs in Figure S2). Additionally, we removed the baseline response measured between -2.25 and 0 s relative to delay onset.

For our multivariate analysis, we implemented an inverted encoding model (IEM). We built the IEM using the average delay-period activation, 5.25-12s following delay onset (average over 9 TRs, each 750ms), of the independent mapping task. The weights trained on these data were then applied to delay period activity of the main experiment, and accordingly reconstructed the channel response amplitude of the stimulus on any given trial.

Effect(s) tested

In the experiment, we performed 3, 2, &1-way ANOVAs as well as t-tests and correlations. All tests, with the exception of the behavioral error and reaction time analyses (Figure 1) were performed using non-parametric shuffling tests. Our two main conditions were 1) distractor absent & 2) distractor present, and for testing with 3 and 2-way ANOVAs, we also considered delay epoch (3 levels, pre-distractor, during distractor, post-distractor), as well as ROI (main text, 7 levels; V1-3,V3AB, hV4, LO1,IPS0/1,IPS2/3, & sPCS). We analyzed our data on a within-subject repeated measures ANOVA basis, where condition labels were shuffled within-participant, and a standard repeated-measures ANOVA was performed and the resulting F-value was stored. We then compared the true F-value generated from a standard, within-subjects repeated measures ANOVA to the distribution of F-values generated from randomly permuting condition labels. We calculated the p-value by determining how many null F-values were less than the true F-value. For t-tests, the identical procedure was followed, and the p-value was calculating by comparing the true T-value to the distribution of null T-values and taking the minimum value for whether T-real was greater than or less than the majority of null distributions (doubled to account for a two-way test).

For the behavioral error-neural error correlations, we performed two separated analyses. First, we performed a trial-by-trial correlation for each participant. We transformed each participant's rho value to a Fisher-Z score, and subjected this group of scores to a t-test. We then randomly shuffled indices within each participant 1000x, computed correlations, and compared our real T-score to these shuffled T-scores, and computed how many shuffled T-scores were greater than our real T-score to determine the one-tailed p-value. Secondarily, we performed a follow-up analysis in which we binned each participants' trials into quartiles, taking one mean per participant per quartile, and performed a correlation on these n_participants x n_quartiles (7x4) datapoints. We report the untransformed rho for these trial-binned correlations, and similarly as above, compare the rho obtained across participants to a shuffled version of the correlation, arriving at a one-tailed p-value.

Specify type of analysis: ☐ Whole brain ☒ ROI-based ☐ Both

Anatomical location(s): ROIs were determined from a separate retinotopic mapping session.

Statistic type for inference
(See Eklund et al. 2016)

Voxels included in our analyses were identified by fitting retinotopic models to each voxel, resulting in a number of fit parameters, including variance explained per voxel. All voxels included in our analyses met at minimum 10% variance explained by the retinotopic model.

Correction

We corrected for multiple comparisons at the ROI level. Voxels were included in all analyses based on variance explained of the best-fit pRF model to the independent retinotopic mapping dataset. No activation maps are used in this study, and so voxelwise correction for multiple comparisons is unnecessary.

Models & analysis

n/a	Involved in the study
☒	☐ Functional and/or effective connectivity
☒	☐ Graph analysis
☐	☒ Multivariate modeling or predictive analysis

Multivariate modeling and predictive analysis

We used an inverted encoding model (IEM) for spatial position (e.g., Sprague et al, 2014; Sprague et al, 2018; eNeuro). For most analyses, we used a 'fixed model' approach whereby we estimated the encoding model using a separate dataset, then used this fixed model to reconstruct WM/distractor representations from each timepoint of the main experiment (see Sprague et al, 2018, eNeuro for a discussion). For Fig. 6, we estimated the model using distractor-present trials on all runs but one, and for each timepoint (or epoch) prior to reconstructing all trials of the held-out run at each timepoint (or epoch). Reconstructions were quantified via their 'fidelity' (Sprague et al, 2016), and compared against a distribution of fidelity values computed with a shuffled model (Fig. 4).
